# The integration of transcriptomic and transgenic analyses reveals the involvement of the SA response pathway in the defense of chrysanthemum against the necrotrophic fungus *Alternaria* sp.

**DOI:** 10.1038/s41438-020-0297-1

**Published:** 2020-06-01

**Authors:** Xiting Zhao, Lingyu Song, Liwei Jiang, Yuting Zhu, Qinghui Gao, Dandan Wang, Jing Xie, Meng Lv, Ping Liu, Mingjun Li

**Affiliations:** 1 College of Life Sciences, Henan Normal University, Xinxiang, 453007 China; 2 Engineering Technology Research Center of Nursing and Utilization of Genuine Chinese Crude Drugs in Henan Province, Xinxiang, 453007 China; 3 College of Mathematics and Information Science, Henan Normal University, Xinxiang, 453007 China

**Keywords:** Plant immunity, Transcriptomics

## Abstract

*Chrysanthemum morifolium* cv. ‘Huaihuang’ has ornamental, edible, medicinal, and tea product uses. However, its field growth, yield, and quality are negatively affected by black spot disease caused by *Alternaria* sp. (Strain: HQJH10092301; GenBank accession number: KF688111). In this study, we transcriptionally and transgenically characterized a new cultivar, ‘Huaiju 2^#^’ (Henan Traditional Chinese Medicine Plant Cultivar identification number: 2016002), which was bred from ‘Huaihuang’ and shows resistance to *Alternaria* sp. Numerous ‘Huaiju 2^#^’ plants were inoculated with *Alternaria* sp. for three or five days. Metabolic analysis showed increases in both salicylic acid (SA) and jasmonic acid (JA) in infected plants compared to the control. Protein activity analysis also revealed a significant increase in defense enzyme activities in infected plants. RNA-Seq of plants infected for 3 or 5 days produced a total of 58.6 GB of clean reads. Among these reads, 16,550 and 13,559 differentially expressed genes (DEGs) were identified in Cm_3 dpi (sample from 3 days post-inoculation labeled as Cm_3 dpi) and Cm_5 dpi (sample from 5 days post-inoculation labeled as Cm_5 dpi), respectively, compared with their controls (Cm_0 d: a mixture samples from 0 d (before inoculation) and those treated with sterile distilled water at 3 dpi and 5 dpi). Gene annotation and cluster analysis of the DEGs revealed a variety of defense responses to *Alternaria* sp. infection, which were characterized by increases in resistance (R) proteins and the reactive oxygen species (ROS), Ca^2+^, mitogen-activated protein kinase (MAPK), and JA signaling pathways. In particular, SA signaling was highly responsive to *Alternaria* sp. infection. The qPCR analysis of 12 DEG candidates supported their differential expression characterized by using the RNA-Seq data. One candidate was *CmNPR1* (*nonexpressor of pathogenesis-related gene 1*), an important positive regulator of SA in systemic acquired resistance (SAR). Overexpression of *CmNPR1* in ‘Huaiju 2^#^’ increased the resistance of transgenic plants to black spot. These findings indicate that the SA response pathway is likely involved in the defense of ‘Huaiju 2^#^’ against *Alternaria* sp. pathogens.

## Introduction

*Chrysanthemum morifolium* produced mainly in Jiaozuo city (ancient Huaiqingfu), Henan Province, China, is referred to as ‘Huaijuhua’ in Chinese. This perennial herb is a traditional Chinese medicine. It is commonly used in various prescriptions from the Chinese pharmacopoeia. It produces three abundant medicinal compounds, 3,5-dicaffeoyl-quinic acid, luteoloside and chlorogenic acids^1^. Other abundant metabolites studied include polysaccharides (CMJA0S2) that have been shown to have inhibitory effects on the growth of pancreatic cancer cells (PANC-1)^2^. In addition to its medicinal uses, ‘Huaijuhua’ is employed as an ornamental plant for landscaping. Its flowers are also used to make tea^3^.

‘Huaijuhua’ is mainly propagated *via* cuttings for different applications. Although this approach is effective, new cuttings are vulnerable to infection by pathogens, such as fungi, bacteria, and viruses. One common disease is black spot disease caused by a fungus pathogen. This disease leads to a severe decrease in plant yield and low-quality products of chrysanthemum flowers, which results in economic loss^4^. To understand the causes of the black spot, we isolated *Alternaria* sp. (Strain: HQJH10092301; GenBank accession number: KF688111) from necrotic plants and used different approaches to demonstrate that it is the pathomycete responsible for the black spot^5^.

To fight against pathogen infection, plants have evolved a multilayered defense mechanism that mainly involves innate passive disease resistance and induced resistance caused by various inducible factors^6–8^. In addition to reacting locally, plants have also evolved a systemic response that establishes an enhanced defensive capacity to protect the plant against subsequent invaders. These systemic responses can be divided into systemic acquired resistance (SAR) and induced systemic resistance (ISR)^9^. SAR is a broad-spectrum resistance response that can be induced by local plant infection with pathogens or treatment with chemical inducers^9–11^. ISR is activated upon the colonization of roots by certain strains of non-pathogenic rhizobacteria^9^. The development of SAR is accompanied by the accumulation of salicylic acid (SA) and the transcription of *PR* genes^11^. The development of ISR is accompanied by the accumulation of jasmonic acid (JA)^9^. Previous studies have shown that SAR effectively inhibits the growth of biotrophic pathogens such as *Pseudomonas syringae*, whereas necrotrophic pathogens are typically more sensitive to ISR^9,12–14^. Moreover, the SA- and JA signaling pathways are antagonistic^15^. For example, SA suppresses JA-regulated basic *PR* gene expression in tobacco^16^. Plants with a silenced *phenyl-ammonia-lyase* gene exhibit reduced levels of SA but higher levels of JA^14^. Spoel et al.^14^ found that SA is a potent inhibitor of JA-dependent defense against necrotrophic fungi. Interestingly, recent research has indicated that SA or JA response pathways are not activated exclusively by biotrophy or necrotrophy, respectively, and that they are synergistic. For instance, using RNA-Seq, Li et al.^4^ showed that JA and SA signaling pathways are both involved in the response of chrysanthemum to infection by the necrotrophic fungus *A. tenuissima*. Mazumder et al.^17^ also found that the necrotrophic fungus *A. brassicicola* can cause the accumulation of SA and inhibit the JA response pathway in the early stage of plant infection. Studies have shown that the SA response pathway is involved in the defense of plants against necrotrophic fungi, but there is still a lack of experimental investigation and evidence.

Nonexpressor of pathogenesis-related genes (NPR) proteins are involved in plant defense. To date, six NPRs have been found in *Arabidopsis thaliana*, designated *AtNPR1*, *AtNPR2*, *AtNPR3*, *AtNPR4*, *AtNPR5*, and *AtNPR6*^18^. Ding et al.^19^ indicated that *AtNPR1* was an important positive regulator of SAR, whereas *AtNPR3* and *AtNPR4* are known negative regulators of SAR. Under steady-state conditions, inactive NPR1 oligomers reside in the cytoplasm. Effector-triggered immunity (ETI) signals facilitate the generation of SA in plants, which results in biphasic redox changes and leads to the phosphorylation of NPR1 and its subsequent monomerization, allowing its translocation into the nucleus to regulate *PR* gene expression through interaction with TGA, WRKY or TCP transcription factors^20,21^. Considerable evidence suggests that NPR1 is the master regulator in plant SAR^18,22^. Overexpression of the *AtNPR1* gene does not significantly change the morphology of *A. thaliana* or increase its resistance to *Xanthomonas campestris* pv. *vesicatoria* and *Ralstonia solanacearum*^23^. The mutation of *NPR1* in plants may disrupt the response of other *PR* genes to SAR^13^. In addition, NPR1 plays an important role in plant ISR that relies on JA response pathways^24^. Plants such as tomato^23^, carrot^25^, cotton^26^, and strawberry^27^ constitutively expressing *AtNPR1* show broad-spectrum resistance to necrotrophic fungi, viruses and bacteria^9,28^.

To overcome the black spot problem, we have developed a new ‘Huaijuhua’ cultivar, ‘Huaiju 2^#^’, which is tolerant to infection by *Alternaria* sp. In this study, we used an integrative approach to understand the mechanisms by which this new cultivar tolerates black spot disease caused by *Alternaria* sp. ‘Huaiju 2^#^’ was propagated *via* cuttings to generate a homogeneous population grown in the greenhouse. Plants at the 15-leaf stage were inoculated with *Alternaria* sp. Symptom development was carefully recorded daily. Samples of infected plants and control plants were collected on two different dates after inoculation. Then, the samples were used for experiments associated with defensive biochemistry and RNA-Seq analysis. Metabolic analysis was carried out to characterize salicylic acid (SA) and jasmonate (JA) patterns. Protein analysis was carried out to understand the activity of defense enzymes. RNA-Seq was used to obtain differentiated transcriptomes associated with responses to infection by *Alternaria* sp. Multiple differentially expressed genes (DEGs) were shown to be associated with pathogen resistance, such as SA-dependent genes. One SA-dependent candidate gene, *CmNPR1*, was overexpressed in ‘Huaiju 2^#^’. The transgenic plants showed enhanced resistance to black spot disease caused by *Alternaria* sp. These data provide useful information for the future breeding of new elite cultivars for obtaining chrysanthemum products.

## Materials and methods

### Plant materials and pathogenic strains

‘Huaiju 2^#^’ (Henan Traditional Chinese Medicine Plant Cultivar identification number: 2016002), a new cultivar of ‘Huaijuhua’, was used in this study. It took us six years to breed this new cultivar from the commercial cultivar ‘Huaihuang’ *via* tissue culture and selection. All breeding experiments were completed at the Engineering Technology Research Center of Nursing and Utilization of Genuine Chinese Crude Drugs in Henan Province, Henan Normal University, Xinxiang, China. In the greenhouse, the plants were grown in 7-cm-diameter pots with a peat-vermiculite (v/v = 1:1) mixture. The growing conditions included a 14 h light/10 h dark light cycle, 20–25 °C temperatures, 40 to 60% relative humidity (RH), and 60 mE·s^−1^·m^−2^ light intensity. When grown in the field, ‘Huaiju 2^#^’ exhibits stronger black spot resistance than ‘Huaihuang’ (Supplementary Fig. S1).

The pathogenic strain used for inoculation was *Alternaria* sp. strain: HQJH10092301 (GenBank accession number: KF688111), a necrotrophic fungus. This strain was isolated from infected ‘Huaihuang’ at the Germplasm Resources Bank of the Wenxian Institute of Agricultural Science in Henan Province, Jiaozuo City, China^5^. Based on the protocol developed by Thomma et al.^29^, the culture conditions for this pathogenic strain were optimized with slight modification^5^. This strain has been maintained for research purposes at the Engineering Technology Research Center of Nursing and Utilization of Genuine Chinese Crude Drugs in Henan Province, Henan Normal University, Xinxiang, China.

### *Alternaria* sp. inoculation and sampling

When plants developed 15 leaves, they were inoculated with *Alternaria* sp. according to the method of Thomma et al.^29^. In brief, activated spores were diluted to a density of 10^7^ spores per milliliter in sterile distilled water (SDW), in which the concentration was measured with a hemocytometer. Three leaves were selected from each plant for inoculation. Five locations on each selected leaf were punctured with a needle (approximately 0.41 mm diameter). Each wounded location was inoculated with 10 μL of spore suspension. On the leaves of the mock plants, each wounded location was inoculated with 10 μL SDW as a control. After inoculation, all inoculated plants were kept in a dark incubation chamber at 25 °C with 100% relative humidity (RH) as reported previously^29^. After 48 h, all plants were transferred to a greenhouse (14 h light/10 h dark light cycle, 20–25 °C, 90–95% RH, 60 mE·s^−1^·m^−2^ light intensity). The phenotypic changes in ‘Huaiju 2^#^’ before and after inoculation with *Alternaria* sp. were recorded by photography. Leaves were harvested from plants at 0 d (before inoculation) and at 3 and 5 days post-inoculation (dpi) with *Alternaria* sp. or SDW (mock), respectively. The leaves were immediately frozen in liquid nitrogen and then stored at −80 °C for biochemical and sequencing experiments.

### Biochemical assays

Frozen leaf samples were used to determine SA and JA contents and the activity of defense enzymes, including phenylalanine ammonia-lyase (PAL), peroxidase (POD), polyphenol oxidase (PPO), ascorbate peroxidase (APX), chitinase (CHT), and glucan endo-1,3-beta-glucosidase (GLU), and RNA-Seq was conducted. SA and JA contents were determined using an enzyme-linked immune sorbent assay (ELISA) in a facility at the ZCI BiO Company (Shanghai, China). This company’s service helped to bind SA and JA to BSA (bovine serum albumin) and then produced the corresponding antibodies. The activity of PAL and POD was measured according to the methods of Liu et al.^30^. The PPO activity assay followed the protocol developed by Hammerschmidt and Kuć^31^. APX activity was measured according to Nakano and Asada^32^. CHT activity was assayed according to Pombo et al.^33^. GLU activity was measured with 3,5-dinitrosalicylic acid (DNS) as reported by Ramamoorthy et al.^34^. For each assay, three biological replicates were performed.

### RNA extraction, cDNA library construction, and sequencing

For RNA-Seq, we used the sequencing service of the Novogene Biological Information Technology Center (Beijing, China). RNA extraction and transcriptome sequencing were conducted in three biological replicates of leaves. In brief, total RNA was extracted from frozen leaf samples using MiniBEST Plant RNA Extraction Kits (Takara). Agarose gels (1%) were used to examine the quality of RNA. RNA purity was checked using a NanoPhotometer® spectrophotometer (IMPLEN). The RNA concentration was measured using a Qubit® RNA Assay Kit in a Qubit® 2.0 Fluorometer (Life Technologies). The quality of the RNA samples (RNA integrity number (RIN) ≥ 6.5, 28 S:18 S > 1.5) was assessed using the RNA Nano 6000 Assay Kit and an Agilent Bioanalyzer 2100 (Agilent Technologies). In total, fifteen high-quality RNA samples from inoculated and mock-treated leaves (three prior to inoculation (0 d), six at 3 dpi, and six at 5 dpi) were prepared for RNA-Seq.

Although there were nine control RNA samples subjected to SDW control inoculation from each of the 0 d, 3 dpi, and 5 dpi time points, we mixed them to form three biological samples for sequencing. A 10 µg RNA sample from each of the 0 d, 3 dpi and 5 dpi samples was mixed to form a single control, which was labeled as Cm_0 d. Accordingly, three biological RNA controls were produced for sequencing. The RNA samples collected 3 dpi and 5 dpi with *Alternaria* sp. were labeled as Cm_3 dpi and Cm_5 dpi, respectively. For cDNA library construction, 1.5 μg of DNA-free RNA was used for reverse transcription to synthesize the first strand of DNA with the NEBNext® Ultra™ RNA Library Prep Kit for Illumina® (NEB, USA). We followed the steps of the manufacturer’s protocol described by Zhong et al.^35^. Finally, nine cDNA libraries (three Cm_0 d, three Cm_3 dpi and three Cm_5 dpi) were constructed for sequencing. RNA-Seq was performed to obtain paired-end reads on the Illumina HiSeq4000 platform. All clean reads generated in this study were deposited in the NCBI Sequence Read Archive database (http://www.ncbi.nlm.nih.gov/sra/) under the project accession number PRJNA448499.

### Filtration and functional analysis of differentially expressed genes (DEGs)

All reads were cleaned with the program SeqPrep (https://github.com/jstjohn/SeqPrep), which removed adapters, poly-N sequences, and low-quality reads were. The resulting clean reads were mapped to the genome of *Alternaria alternata* ATCC 34957 (GenBank accession number: LMXP00000000), so that reads belonging to *Alternaria* pathogen sequences could be removed from the total reads. The pathogen sequence-free reads were *de novo* assembled to obtain contigs using the Trinity program. All contigs were analyzed with RSEM software to map the clean reads of each sample to the transcriptional reference sequence obtained *via* Trinity splicing^36^. The FPKM (fragments per kilobase of exon per million fragments mapped) value of each unigene was calculated with Cufflinks software to determine its gene expression level according to an established protocol^37^. Raw counts were obtained for each unigene and subsequently subjected to analysis with the DESeq R package to identify DEGs between Cm_0 d and Cm_3 dpi or Cm_5 dpi. The parameters used to identify DEGs included a cutoff of a | log2foldchange| > 1 and a false discovery rate (FDR) < 0.05^38^. Gene Ontology (GO) enrichment analysis and Kyoto Encyclopedia of Genes and Genomes (KEGG) pathway analysis of the DEGs were implemented using the GOseq package^39^ and KOBAS software^40^, respectively. A gene expression heatmap of the DEGs was performed using R-Studio v8.8.171971.

### Quantitative real-time PCR

To verify the transcriptome sequencing results, we randomly selected 12 DEGs related to disease resistance for verification by qPCR. Primer pairs were designed with Primer Premier 5.0 (http://www.premierbiosoft.com/) based on the RNA-Seq results. The resulting primer pairs were 18–24 bp in length, with Tms of 58–62 °C, and GC contents of 50–60%, and they amplified 100–200 bp-long fragments. All primer pairs were specific to one of the 12 selected DEGs (Table S1).

For qPCR experiments, we used the same nine biological RNA samples for RNA-Seq, three biological replicates for the control, three for 3 dpi, and three for 5 dpi as described above. RNA was reverse transcribed into first strand cDNA using the HiScriptTM Q Select RT SuperMix Kit (Vazyme, USA). qPCR was performed in a LightCycler® 96 real-time PCR system (Roche, Switzerland) with AceQ® Universal SYBR® Green Master Mix (Vazyme, USA), and the reaction system and procedure followed the reagent specifications. To analyze the qPCR data, the expression of candidate genes was normalized to that of the *CmUBI* gene, a housekeeping gene in chrysanthemum. Melting curve analysis was performed, and the absence of non-specific products of primer pairs was verified (Supplementary Fig. S5)^41^. The relative expression level of genes was calculated using the 2^−ΔΔCt^ method^42^. The experiment was performed with at least three independent replicates.

### Generation of transgenic plants for pathogen inoculation

Through sequencing, we identified a pathogen-resistant cDNA, *CmNPR1*. Its ORF was isolated from ‘Huaiju 2^#^’ and cloned into the Super1300-35S-GFP binary vector (kindly donated by Professor Gao Junping of China Agricultural University). The resulting recombinant binary vector was introduced into the *Agrobacterium* strain, and the *Agrobacterium* strain harboring our binary vector was used to transform ‘Huaiju 2^#^’ with a leaf disk transformation method according to a reported protocol^43^. Multiple transgenic plants were obtained and grown in pots for pathogen inoculation as described below.

### Necrotrophic *Alternaria* sp. resistance assay of transgenic plants

As described above, leaves from plants at the 9–13 leaf stage from the wild-type (WT) and transgenic (*35* *S::CmNPR1 #4*) lines were inoculated with *Alternaria* sp. Three independent experiments were performed with 30 plants per line. The phenotypic changes in ‘Huaiju 2^#^’ were also recorded as described above. The sampling time points were 0 d, 3 and 5 dpi. Harvested leaves were quickly frozen in liquid nitrogen and stored at −80 °C. Subsequently, the activities of PAL, superoxide dismutase (SOD), POD, and APX were analyzed as reported by Liu et al.^30^. CAT activity was measured as described by Kang et al.^44^. GR activity was measured as described by Ge et al.^45^. Additionally, qPCR was performed to analyze the expression of three SA response pathway genes and three defense genes. The SA response pathway genes were *CmTGA1*, *CmTGA5*, and *CmPR5*, and the plant defense genes were *CmPAL*, *CmPOD*, and *CmCHT*.

### Statistical analysis

Data are presented as the means ± standard errors (SE). Differences in outcomes between the treatments were evaluated in Excel 2010 (Microsoft, USA) and SPSS 13.0 software (IBM, USA). Independent-samples Student’s *t*-tests and one-way ANOVA were performed to evaluate statistical significance.

## Results

### The effect of *Alternaria* sp. on the phenotype and biochemistry of ‘Huaiju 2#’

**Fig. 1:**
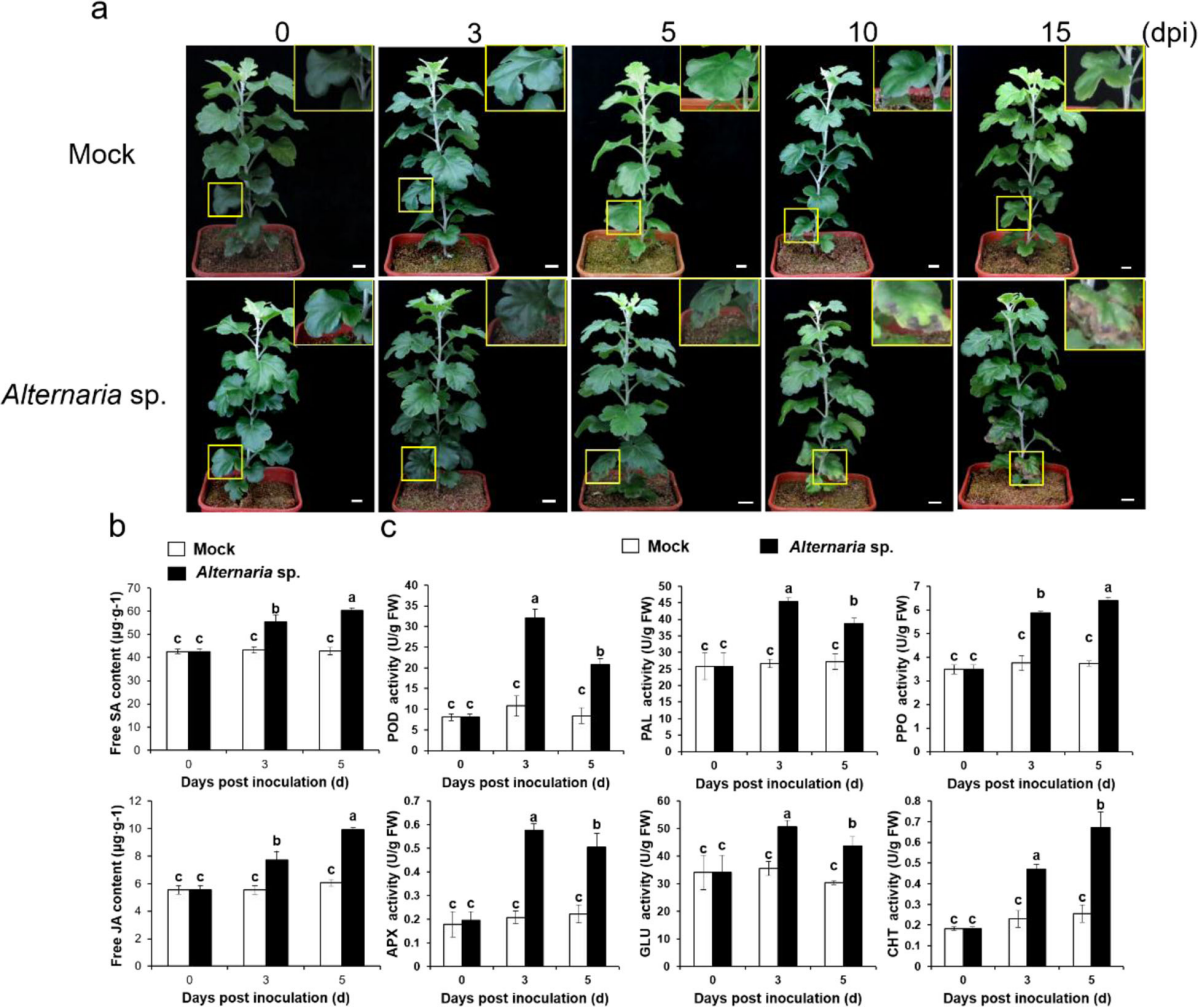
Changes in phenotype (**a**) hormone content (**b**), and defense enzyme activities (**c**) after ‘Huaiju 2^#^’ was inoculated with sterile distilled water (SDW, mock) or *Alternaria* sp. The inserted yellow box in the top right corner is a magnified image of the leaf selected from the bottom of the plants highlighted by a yellow frame. The data are the means of three biological repeats. Error bars indicate SEs. Letters above the histogram indicate statistically significant differences among different lines (*P* < 0.05) determined using one-way ANOVA. Scale bars: 1 cm

We observed and recorded phenotypic changes in ‘Huaiju 2^#^’ to investigate the effect of *Alternaria* sp. infection on ‘Huaiju 2^#^’ growth. Although this new variety showed better tolerance to this fungus than its parent plants^5^, after 10 days of inoculation, the leaves developed typical disease spots, which continued to grow larger to form apparent necrotic symptoms after 15 days of inoculation. In contrast, this pathogenic response was not observed on control leaves treated with the mock SDW control (Fig. 1a). To determine whether the SA or JA pathway in the leaves of ‘Huaiju 2^#^’ responded to *Alternaria* sp., an ELISA was performed. The resulting data showed that the levels of SA and JA were increased significantly in inoculated leaves compared to their levels in controls in the first 5 days (Fig. 1b). Additionally, enzymatic assays were performed to examine whether PAL, POD, PPO, APX, GLU, and CHT were responsive to *Alternaria* sp. infection. The results showed that the activities of all of these enzymes were significantly higher in leaves infected with *Alternaria* sp. than in control leaves treated with SDW (Fig. 1c). These data indicate that the defense system in ‘Huaiju 2^#^’ is responsive to *Alternaria* sp. infection.

### RNA-Seq analysis of gene expression

We next conducted the RNA-Seq analysis of *Alternaria* sp.-infected leaves and mock control leaves. A total of approximately 58.6 GB clean reads were generated from nine biological samples, including six infected and three control samples. The average Q20 and Q30 values of the raw reads were 94.93% and 90.15%, respectively, indicating high-quality reads (Table S2). Approximately 70% of the reads were mapped to the reference genome sequences obtained by Trinity splicing (Table S3). Further correlation evaluation showed that the gene expression levels between samples were consistent with the overall quality of the RNA-Seq results (Supplementary Fig. S2), indicating the reliability of the sampling and experimental design. To obtain a comprehensive view of the gene expression profile associated with the response of ‘Huaiju 2^#^’ to *Alternaria* sp. infection, we used the DESeq R package to identify DEGs (Supplementary Fig. S3a–c). Based on the filtering parameters of padj < 0.05 and a | log2foldchange | > l, the expression of 16,550 and 13,559 genes was found to differ significantly in Cm_3 dpi vs Cm_0 d and Cm_5 dpi vs Cm_0 d, respectively. In addition, 7922 DEGs were identified in the comparison of Cm_5 dpi vs Cm_3 dpi (Supplementary Fig. S3d).

**Fig. 2:**
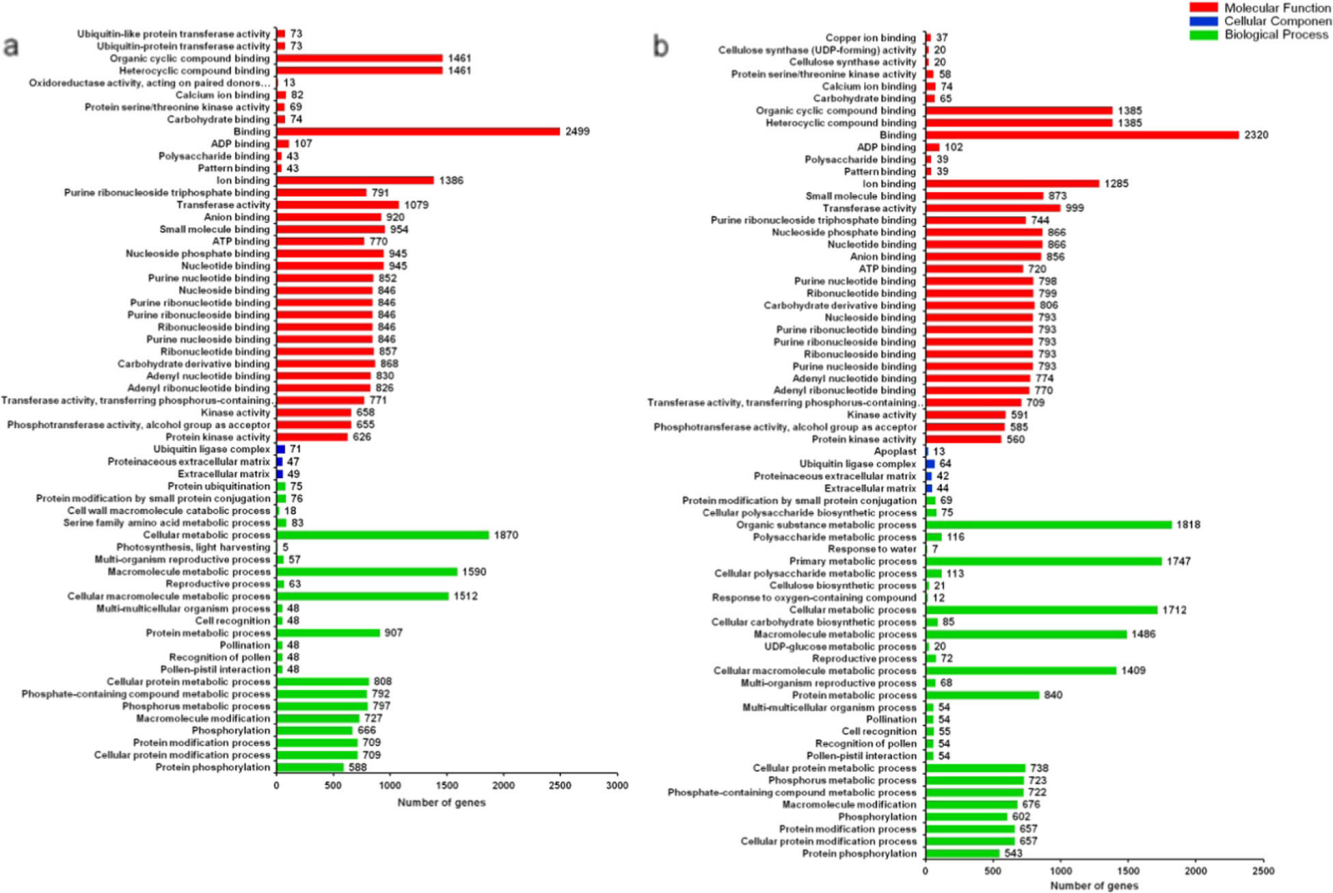
GO analysis based on upregulated DEGs in Cm_3 dpi vs Cm_0 d (**a**) and Cm_5 dpi vs Cm_0 d (**b**)

To understand the functions of the DEGs associated with *Alternaria* sp. infection, sequences from Cm_0 d, 6206 DEGs from Cm_3 dpi and 5455 DEGs Cm_5 dpi were annotated using GOseq. This annotation resulted in three major categories: biological processes, cellular components, and molecular functions. The analysis of the biological process category revealed that 1870 upregulated unigenes from Cm_3 dpi vs Cm_0 d were associated with the ‘cellular metabolic process’ term and that 1818 upregulated unigenes from Cm_5 dpi vs Cm_0 d were associated with the ‘organic substance metabolic process’ term. In the category of cellular components, the ‘ubiquitin ligase complex’ subclass was the most abundant in Cm_3 dpi vs Cm_0 d and Cm_5 dpi vs Cm_0 d. In the category of molecular functions, the maximum number of categories showing upregulated DEG enrichment was related to ‘binding’, and the most significant subclass was ‘protein kinase activity’ in Cm_3 dpi vs Cm_0 d and Cm_5 dpi vs Cm_0 d (Fig. 2).

**Fig. 3:**
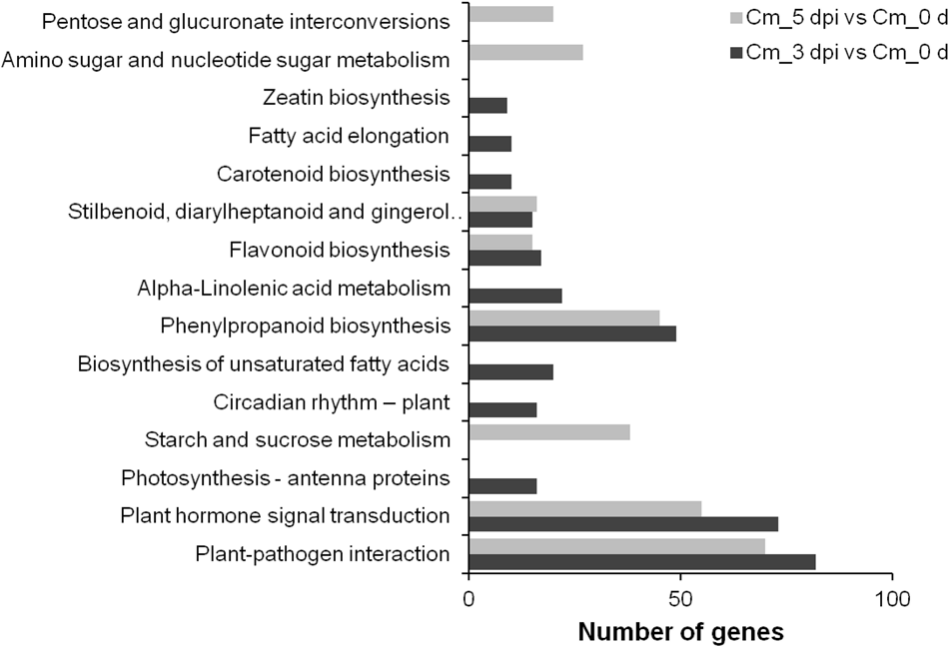
DEGs enriched in different KEGG pathways

To better understand the metabolic or signal transduction pathways activated by *Alternaria* sp. infection, the sequences were annotated with KOBAS. Compared to Cm_0 d, 571 DEGs were assigned to 13 pathways in Cm_3 dpi, and 430 DEGs were assigned to 10 pathways in Cm_5 dpi. Among these DEGs, genes involved in ‘plant-pathogen interaction’ were the most abundant, followed by genes involved ‘plant hormone signal transduction’, ‘phenylpropanoid biosynthesis’, and then ‘flavonoid biosynthesis’ (Fig. 3).

**Fig. 4:**
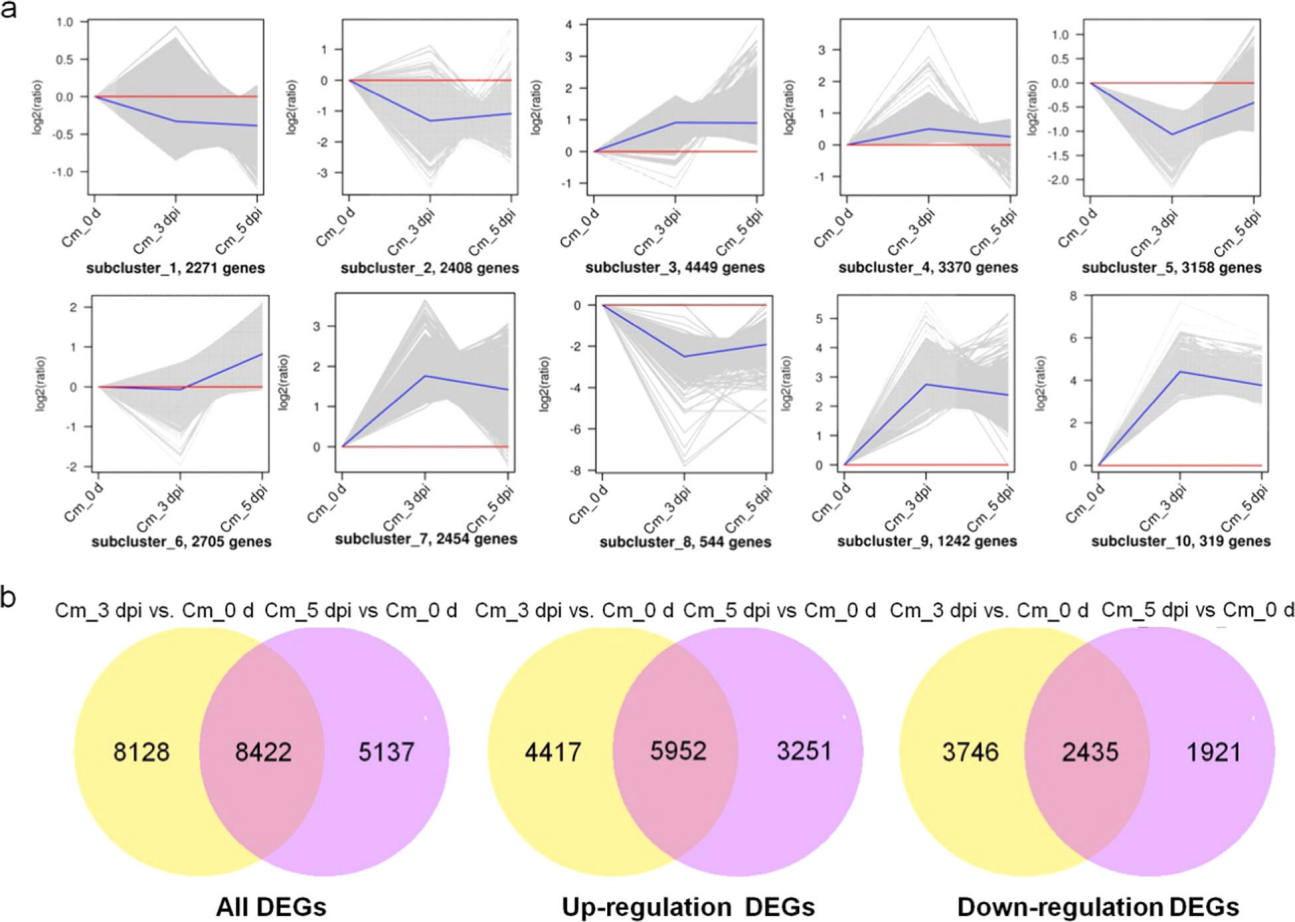
Analysis of continuously upregulated DEGs. **a** Cluster analysis of DEGs; **b** Venn diagrams of continuously upregulated DEGs. The gray line represents the relative expression of genes in a cluster in different samples, and the blue line represents the average relative expression of all genes in the cluster in different samples. The overlapping sections of the circles represent common DEGs between the two combinations

To determine whether the DEGs were associated with different time points, gene expression clustering was employed to identify genes with similar expression patterns. Through this analysis, we obtained 10 clusters of DEGs, among which the expression of the DEGs in subcluster_3 was relatively high and stable, while the DEGs in the other clusters were expressed dynamically (Fig. 4a). To understand genes specifically expressed at one time point, DEGs were analyzed to create a Venn diagram, which showed 5952 up- and 2435 downregulated genes. In particular, 8128 genes were specifically expressed in Cm_3 dpi vs Cm_0 d, including 4417 up- and 3746 downregulated genes. A total of 5137 genes were specifically expressed in Cm_5 dpi vs Cm_0 d, including 4417 up- and 3746 downregulated genes (Fig. 4b). To understand the functions of continuously upregulated DEGs, we also performed KEGG pathway enrichment analysis and obtained a scatter diagram. The continuously upregulated DEGs often belonged to the ‘plant-pathogen interaction’, ‘starch and sucrose metabolism’, ‘plant hormone signal transduction’, ‘phenylpropanoid biosynthesis’, and ‘flavonoid biosynthesis’ categories (Supplementary Fig. S4).

**Fig. 5:**
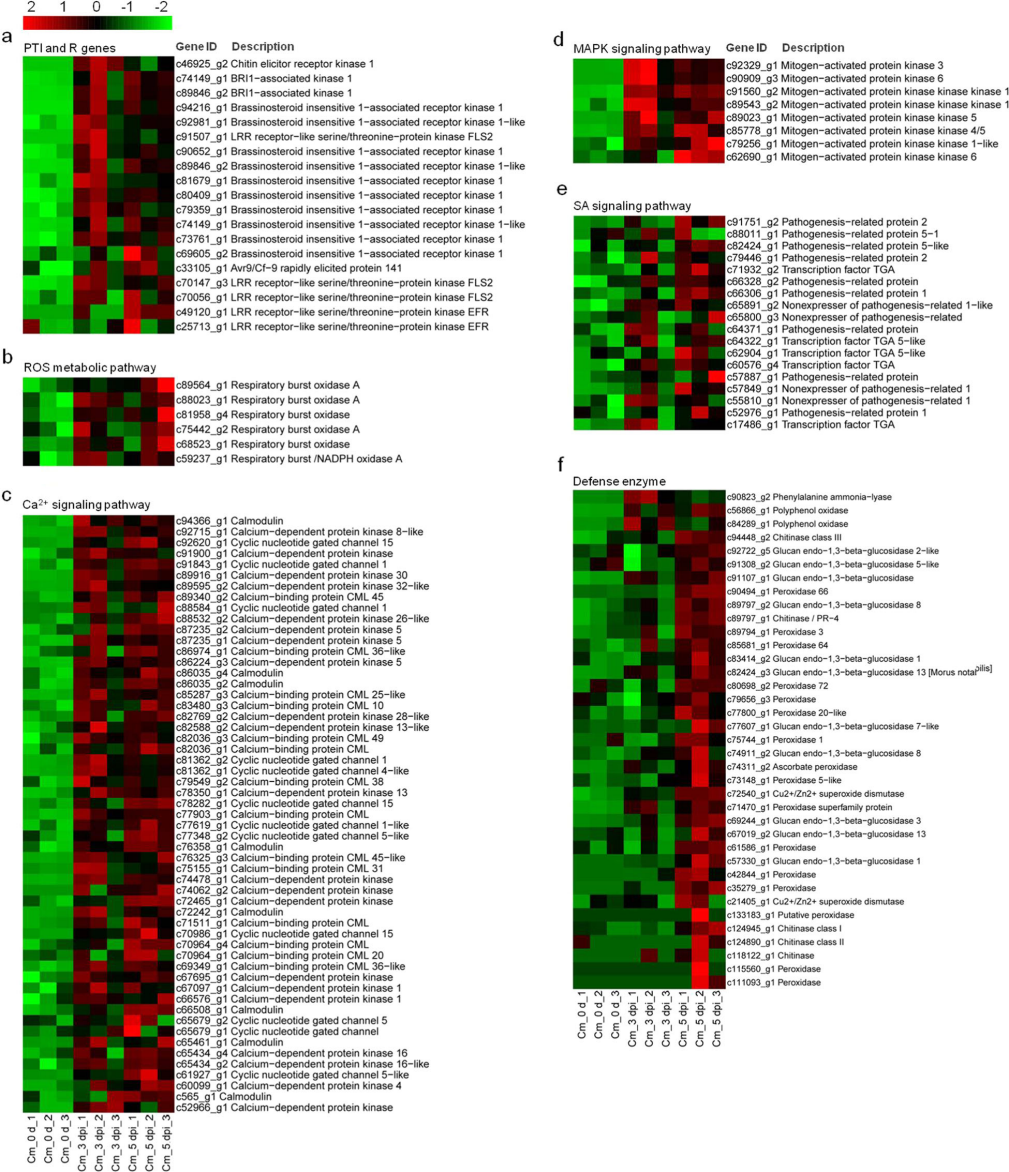
Heatmap of genes continuously upregulated in ‘Huaiju 2#’ in response to *Alternaria* sp. infection. The bar represents the scale of the expression levels for each gene (FPKM) in the different treatments, as indicated by red/green rectangles. Genes in red show upregulation, and those in green show downregulation. **a** PTI and R genes, **b** ROS metabolic pathway, **c** Ca^2+^ signaling pathway, **d** MAPK signaling pathway, **e** SA signaling pathway, **f** Defense enzyme

**Fig. 6:**
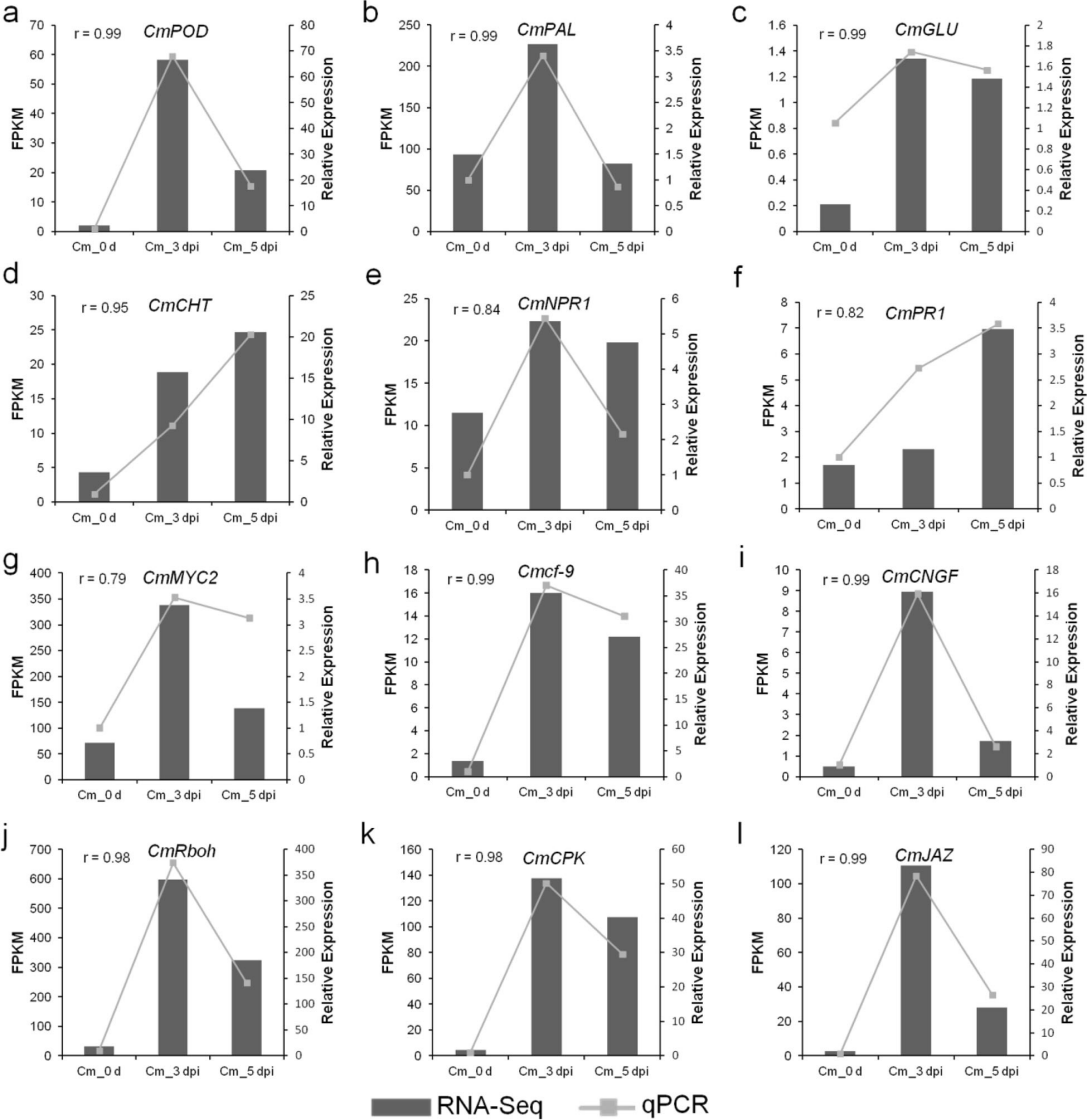
The expression levels of 12 DEGs identified in ‘Huaiju 2#’ in association with the response to *Alternaria* sp. infection. The correlations between the expression profiles of the 12 DEGs were determined by RNA-Seq and qPCR analysis. The left *y*-axes show FPKM values determined by RNA-Seq, and the right *y*-axes show relative expression levels determined by qPCR. *CmUBI* was used as a reference gene. The *r* value between the RNA-Seq and qPCR results is listed in the left corner of each figure representing gene expression. **a** *CmPOD*, **b** *CmPAL*, **c** *CmGLU*, **d** *CmCHT*, **e** *CmNPR1*, **f** *CmPR1*, **g** *CmWYC2*, **h** *Cmcf-9*, **i** *CmCNGF*, **j** *CmRboh*, **k** *CmCPK*, **l** *CmJAZ*

Heatmaps of subcluster_3 were developed to better understand the key DEGs associated with the resistance of ‘Huaiju 2^#^’ to *Alternaria* sp. The resulting heatmaps showed continuously upregulated DEGs involved in plant-pathogen interactions. Based on their functional annotation, these genes included 18 PAMP-triggered immunity (PTI) genes, one R gene (Figs. 5a), 6 reactive oxygen species (ROS) metabolic pathway genes (Fig. 5b), 55 Ca^2+^ signaling pathway genes (Fig. 5c), and 8 mitogen-activated protein kinases (MAPK) signaling pathway unigenes (Fig. 5d). Eighteen DEGs involved in the SA response pathway were obtained, including 9 *PRs*, 5 transcription factor TGA genes, and 4 *NPR1* unigenes (Fig. 5e). In addition, 37 defense enzyme genes, including 15 *PODs*, one *PAL*, 11 *GLUs*, 5 *CHTs*, 2 *PPOs*, one *APX*, and 2 *SOD* unigenes, are shown in the heatmap (Fig. 5f).

### Validation of candidate DEGs with qPCR analysis

To validate the reliability of DEGs obtained from RNA-Seq analyses, the expression levels of 12 candidate genes were analyzed with qPCR. These genes included 4 defense enzyme genes (*CmPAL*, *CmPOD*, *CmGLU*, and *CmCHT*), 2 SA response signaling pathway genes (*CmNPR1* and *CmPR1*), 2 Ca^2+^ signaling pathway genes (*CmCPK* and *CmCNGF*), one ROS metabolic pathway gene (*CmRboh*), one PTI gene (*CmCf-9*) and 2 JA signaling pathway genes (*CmMYC2* and *CmJAZ*). The correlation coefficients (r) between the RNA-Seq and qPCR results were calculated for these DEGs. The results showed that the qPCR data were closely correlated with the RNA-Seq data, indicating that the RNA-Seq data were reliable. Additionally, the qPCR data showed that *CmMYC2* and *CmJAZ*, involved in the JA signaling pathway, and *CmNPR1* and *CmPR1*, associated with the SA signaling pathway, were upregulated after ‘Huaiju 2^#^’ was inoculated with *Alternaria* sp. (Fig. 6).

### Overexpression of *CmNPR1* enhances resistance to *Alternaria* sp. infection

**Fig. 7:**
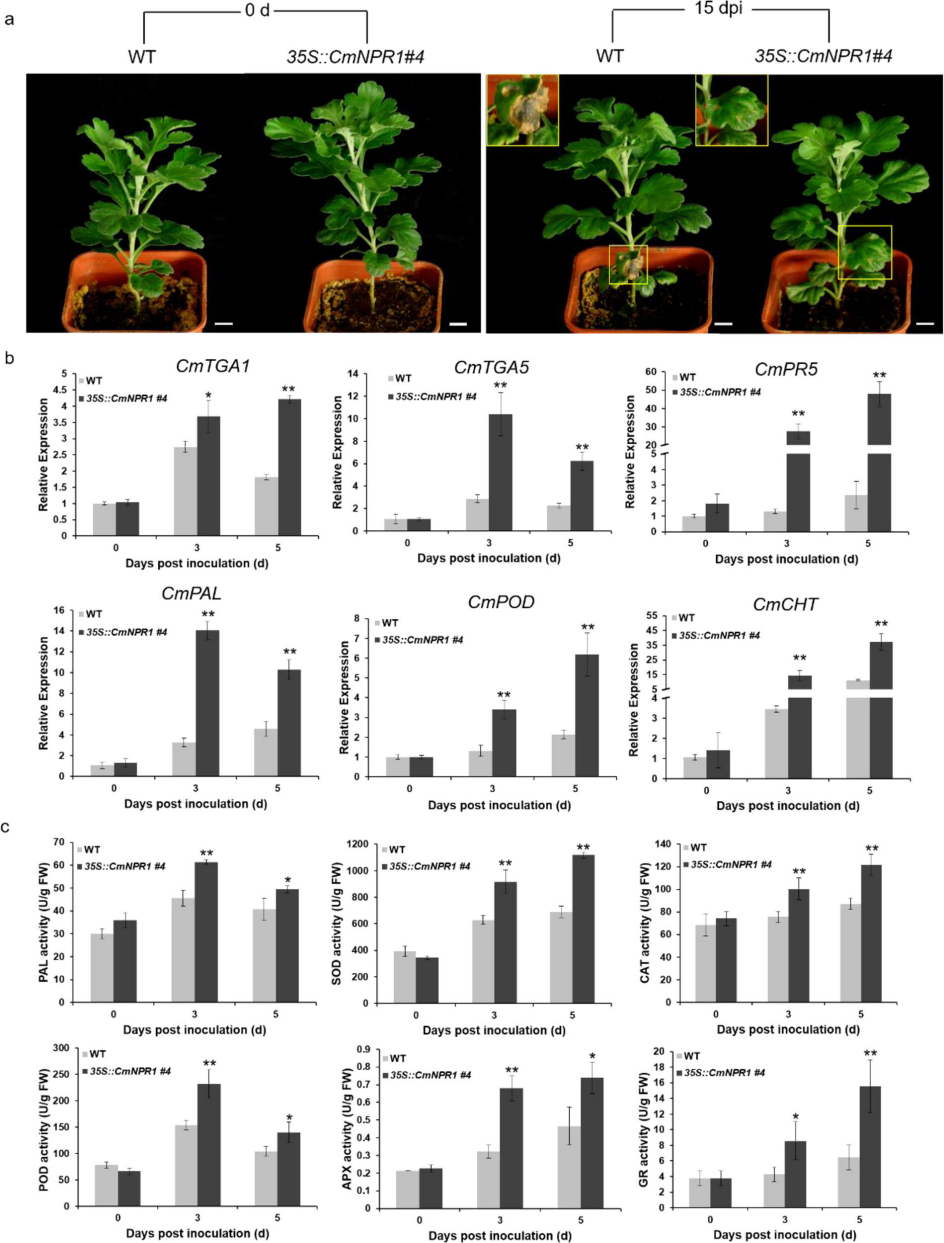
Overexpression of *CmNPR1* increased ‘Huaiju 2#’ resistance to black spot. **a** *Alternaria* sp. infection phenotypes on inoculated leaves of WT and *35S::CmNPR1 #4* plants; **b** Expression profiles of three genes of the SA response pathway and three genes involved in plant defense in WT and *35S::CmNPR1 #4* plants after inoculation for 3 or 5 days; **c** Estimation of the activity of six defense enzymes in WT and *35S::CmNPR1 #4* plants after inoculation for 3 or 5 days. The data are the means of three repeats. Error bars indicate SEs. Asterisks represent significant differences between WT and *35S::CmNPR1 #4* (Student’s *t*-test, **P < 0.01, *P < 0.05)

To determine whether the increased expression of genes of the SA response pathway was associated with the tolerance of ‘Huaiju 2^#^’ to *Alternaria* sp., we cloned *CmNPR1*, an upregulated DEG encoding a transcription co-factor related to the SA signaling pathway, and used the 35 S promoter to overexpress it in ‘Huaiju 2^#^’. Based on selection with hygromycin B (HmB) resistance and verification by semiquantitative PCR and qPCR, eleven positive transgenic lines were generated from the genetic transformation of ‘Huaiju 2^#^’. Additionally, based on the expression level of the transgene, three transformants, *35* *S::CmNPR1 #17* (lower overexpression), *35* *S::CmNPR1 #10* (middle overexpression) and *35* *S::CmNPR1 #4* (higher overexpression) (Supplementary Fig. S6a,b), were selected to evaluate their resistance to *Alternaria* sp. infection using the method described above. The results showed that the three lines exhibited strong resistance to *Alternaria* sp. infection and showed reduced black spot development (Fig. 7, Supplementary Fig. S7). In particular, the *35* *S::CmNPR1 #4* line showed the weakest symptoms (Fig. 7a), indicating that the higher the expression of *CmNPR1*, the better the resistance. Furthermore, we performed qPCR to characterize the expression profiles of three genes involved in the SA response pathway and three genes associated with plant defense in transgenic plants. The results showed that the expression levels of the three SA response pathway genes, *CmTGA1*, *CmTGA5*, and *CmPR5*, were significantly higher in *35* *S::CmNPR1 #4* than in the wild-type controls at 3 and 5 dpi. The expression levels of the three defense genes, *CmPAL*, *CmPOD*, and *CmCHT*, were also significantly higher in *35* *S::CmNPR1 #4* than in WT at these two time points. Further enzymatic assays showed that the activities of the three enzymes were significantly increased in *35* *S::CmNPR1 #4* compared to those in wild-type control plants (Fig. 7c). Similar results were obtained in the *35* *S::CmNPR1 #17* line (with lower overexpression) and the *35* *S::CmNPR1 #10* line (with intermediate overexpression) (Supplementary Fig. S7). These findings indicated that the overexpression of *CmNPR1* in ‘Huaiju 2^#^’ increased the activity of the SA response pathway associated with resistance to black spot.

## Discussion

Our study provides useful information for breeding black spot-resistant *Chrysanthemum* cultivars. As described above, *Chrysanthemum* ‘Huaihuang’ is an important economic crop in China. However, its yield and quality are affected by multiple factors. Black spot caused by *Alternaria* sp.^5^ is severe in this plant and causes major economic losses to farmers^4^. To improve plant crop resistance to black spot diseases, our past efforts have included the breeding of a new cultivar, ‘Huaiju 2^#^’ (Henan Traditional Chinese Medicine Plant Cultivar identification number: 2016002), with higher tolerance to *Alternaria* sp. infection than its parent cultivar ‘Huaihuang’.

In this study, to understand the mechanism of ‘Huaiju 2^#^’ tolerance to *Alternaria* sp., RNA-Seq was employed to analyze DEGs in ‘Huaiju 2^#^’ at different time points after inoculation with *Alternaria* sp. Our findings revealed a large number of DEGs that were putatively related to the response of ‘Huaiju 2^#^’ to the necrotroph *Alternaria* sp. Numerous DEGs are involved in various defense responses that may be mediated by resistance (R) proteins and ROS, Ca^2+^, MAPK, SA (Fig. 5), and JA signaling pathways (Supplementary Table S4). All of these transcriptomic data indicate that multiple processes in ‘Huaiju 2^#^’ are associated with plant defense against pathogens, in line with the fact that plants have evolved a complex defense mechanism^6,7^. In general, in the first stage of infection, fungal pathogens secrete PAMPs that are recognized by pattern recognition receptors (PRRs) on host cell surfaces, thus eliciting PTI^6,46,47^. The activation of PTI results in a series of cellular responses, including the generation of ROS, changes in cytosolic ion flux, cascade activation of calcium-dependent or MAPK, and enhancement of physical barriers^6,47–49^. In this study, 18 PTI (Fig. 5a), 55 Ca^2+^ signaling pathway (Fig. 5c), and 8 MAPK signaling pathway unigenes (Fig. 5d) were found to be upregulated in ‘Huaiju 2^#^ in response to the necrotrophic fungus *Alternaria* sp. Fungal pathogens produce an arsenal of effector proteins to interfere with the recognition of PAMPs by PRRs and facilitate pathogen colonization^7,50^, while plants encode R proteins to activate ETI, which produces hypersensitive responses (HR) at the site of infection, causing cell death to prevent the further spread of pathogens^7,51^. Our RNA-Seq data showed that two leucine-rich repeat (LRR) receptors (EFR and FLS2) (Figs. 5a) and 6 ROS metabolic pathway unigenes (Fig. 5b) were significantly upregulated after *Alternaria* sp. infection. This result suggests that the invasion of *Alternaria* sp. also triggered ETI in ‘Huaiju 2^#^’.

Research has shown that the SA response pathway is associated with plant resistance to biotrophic and hemibiotrophic pathogens, while the JA signaling pathway plays an important role in resisting the invasion of necrotrophic pathogens^52–54^. In our study, ‘Huaiju 2^#^’ was inoculated with *Alternaria* sp. a typical necrotrophic pathogen. The analysis of DEGs revealed not only the upregulation of JA signaling pathway genes, including *CmLOX*, *CmAOS*, *CmOPR*, *CmJAR1*, *CmJAZ*, *CmMYC2* (Table S4), and *CmLOX2* (Supplementary Fig. S8) but also the increased expression of several key genes involved in the SA biosynthesis and signaling pathways, including *CmPAL*, *CmNPR1*, *CmTGA*, and *CmPRs* (Fig. 5e). In addition, the SA content significantly increased after ‘Huaiju 2^#^’ was inoculated with *Alternaria* sp. for 3 or 5 days (Fig. 1b).

*NPR1* is an interesting gene associated with the SA response pathway^13,52^. One study reported that the expression of *BjNPR1* increased the resistance of *Brassica juncea* to *Alternaria brassicae*^52^. *AtNPR1* and its orthologs have been demonstrated to increase resistance to necrotrophic and biotrophic fungal, viral, and bacterial pathogens in a number of plants^23,25,55,56^. In our study, we obtained *CmNPR1* transgenic ‘Huaiju 2^#^’ plants, which showed increased expression levels of three SA response pathway genes (*CmTGA1*, *CmTGA5*, and *CmPR5*) and resistance to black spot (Fig. 7). SA is mainly synthesized by two pathways: the PAL pathway and the isochorismate synthesis pathway^57^. In our study, we showed that *CmPAL* was upregulated after infection with *Alternaria* sp. These data imply that SA may be synthesized *via* the PAL pathway in ‘Huaiju 2^#^’ (Fig. 5f).

**Fig. 8:**
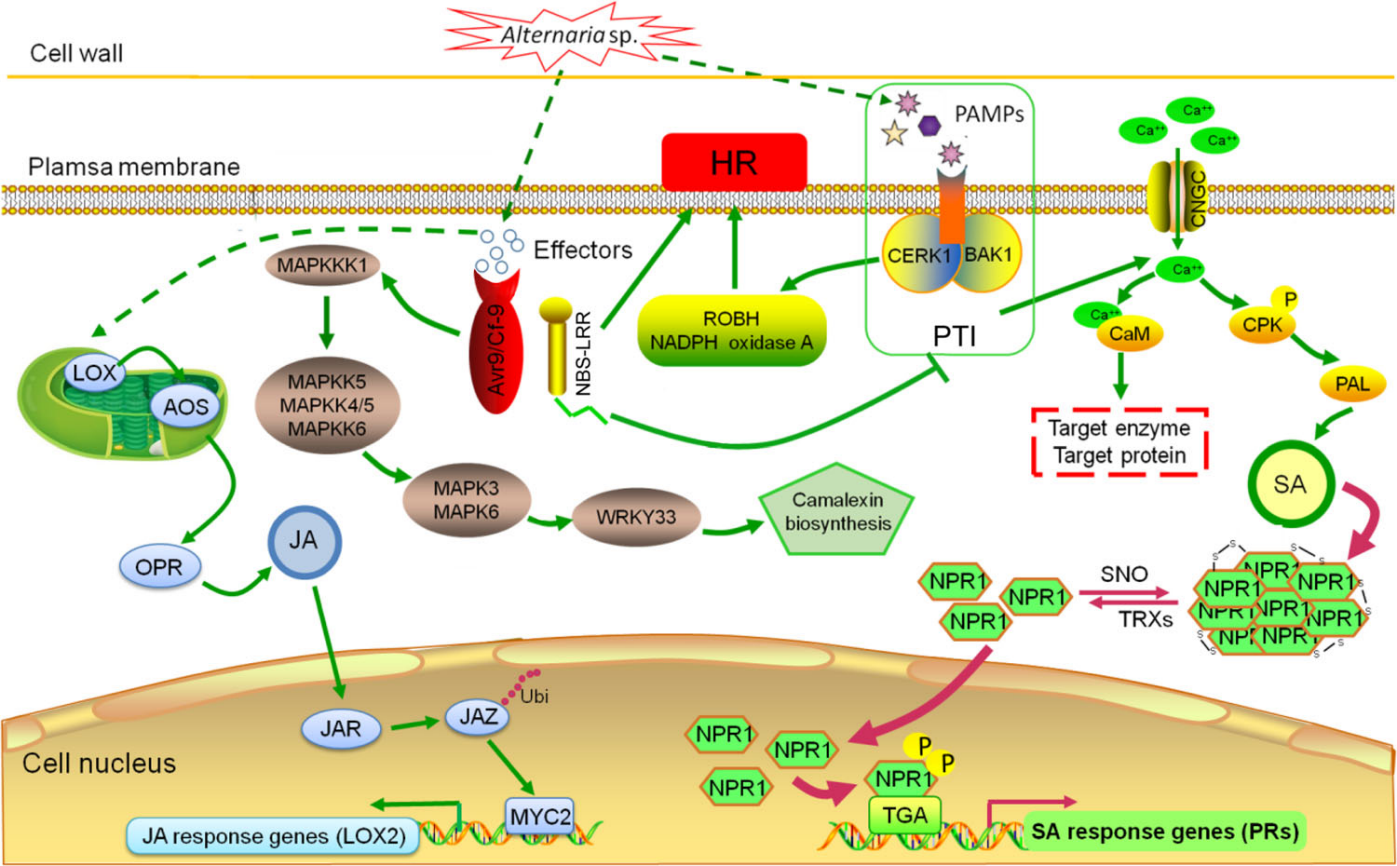
Hypothetical model of the mechanism of ‘Huaiju 2#’ tolerance to *Alternaria* sp. A dotted line represents an unknown to be studied, a solid green line represents the information found by the RNA-Seq, and a solid rose red line represents the information verified by experiments in this work

Based on our data, we propose a hypothetical model for interpreting ‘Huaiju 2^#^’ defense against *Alternaria* sp. (Fig. 8). This model includes genes involved in the JA signaling, SA response, JA biosynthesis, SA biosynthesis, PTI and defense-related pathways and other genes that exist in the genome of ‘Huaiju 2^#^’. When the plant is infected by the necrotrophic *Alternaria* sp. pathogen, the expression of these genes that increase tolerance to black spot is activated. The overexpression of *CmNPR1* in ‘Huaiju 2^#^’ increases the resistance of the plant to *Alternaria* sp. infection.

In conclusion, more than 58.6 GB of clean reads were generated through RNA-Seq. A total of 16,550 and 13,559 DEGs were obtained in the comparisons of Cm_3 dpi and Cm_5 dpi, respectively, compare to Cm_0 d. Functional annotation and cluster analysis of the DEGs showed that a variety of defense responses mediated by R proteins, ROS signaling, Ca^2+^ signaling, MAPK signaling, and SA signaling were activated in the ‘Huaiju 2^#^’ response to *Alternaria* sp. Overexpression of *CmNPR1* increased plant resistance to *Alternaria* sp. Our results suggest that the SA response pathway and other signaling pathways participate in the response of ‘Huaiju 2^#^’ to the necrotrophic fungus *Alternaria* sp.

## Supplementary information

An integration of transcriptomic and transgenic analysis reveals an involvement of SA response pathway in the defense of chrysanthemum to the necrotrophic fungus Alternaria sp
