# Supplementary material for: The integration of transcriptomic and transgenic analyses reveals the involvement of the SA response pathway in the defense of chrysanthemum against the necrotrophic fungus Alternaria sp
Source: Hortic Res. 2020 Jun 1;7:80. doi: 10.1038/s41438-020-0297-1 (PMC7261770; doi:10.1038/s41438-020-0297-1)
Supplement: Supplementary file 1 — An integration of transcriptomic and transgenic analysis reveals an involvement of SA response pathway in the defense of chrysanthemum to the necrotrophic fungus Alternaria sp [file 41438_2020_297_MOESM1_ESM.doc]

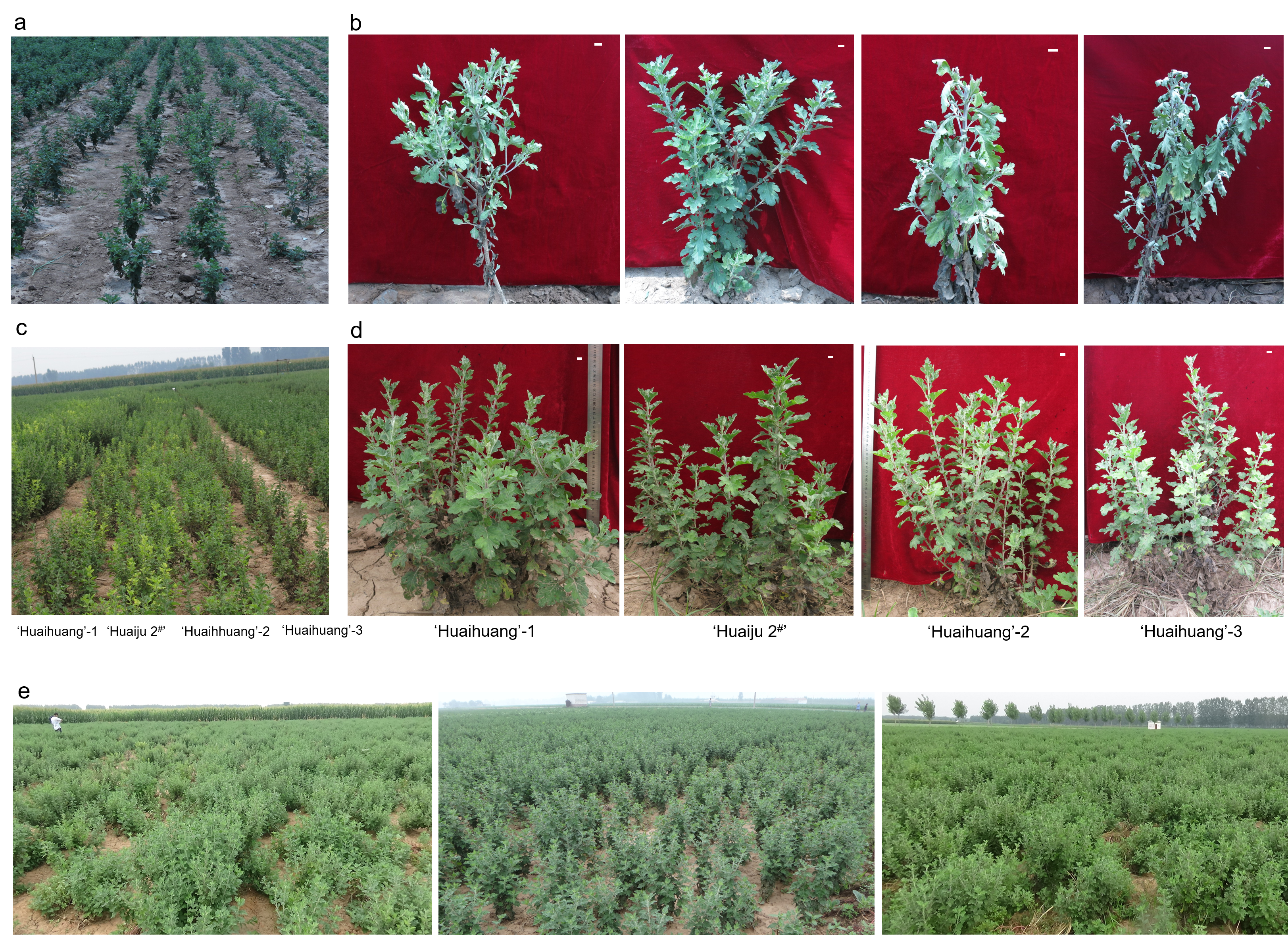


**Supplementary Fig. S1** Comparison of the field characters of ‘Huaiju 2^#^’ and ‘Huaihuang’. The original cultivar (‘Huaihuang’) showed significant symptoms of black spot disease at 75 d after transplant, while ‘Huaiju 2^#^’ didn’t show symptoms until 135 d after transplant. a and b. Field performance of ‘Huaiju 2^#^’ and ‘Huaihuang’ at 75 d after transplant. c and d. Field performance of ‘Huaiju 2^#^’ and ‘Huaihuang’ at 135 d after transplant. e. Different field experiments of ‘Huaiju 2^#^’ at 135 d after transplant. Scale bars: 1 cm


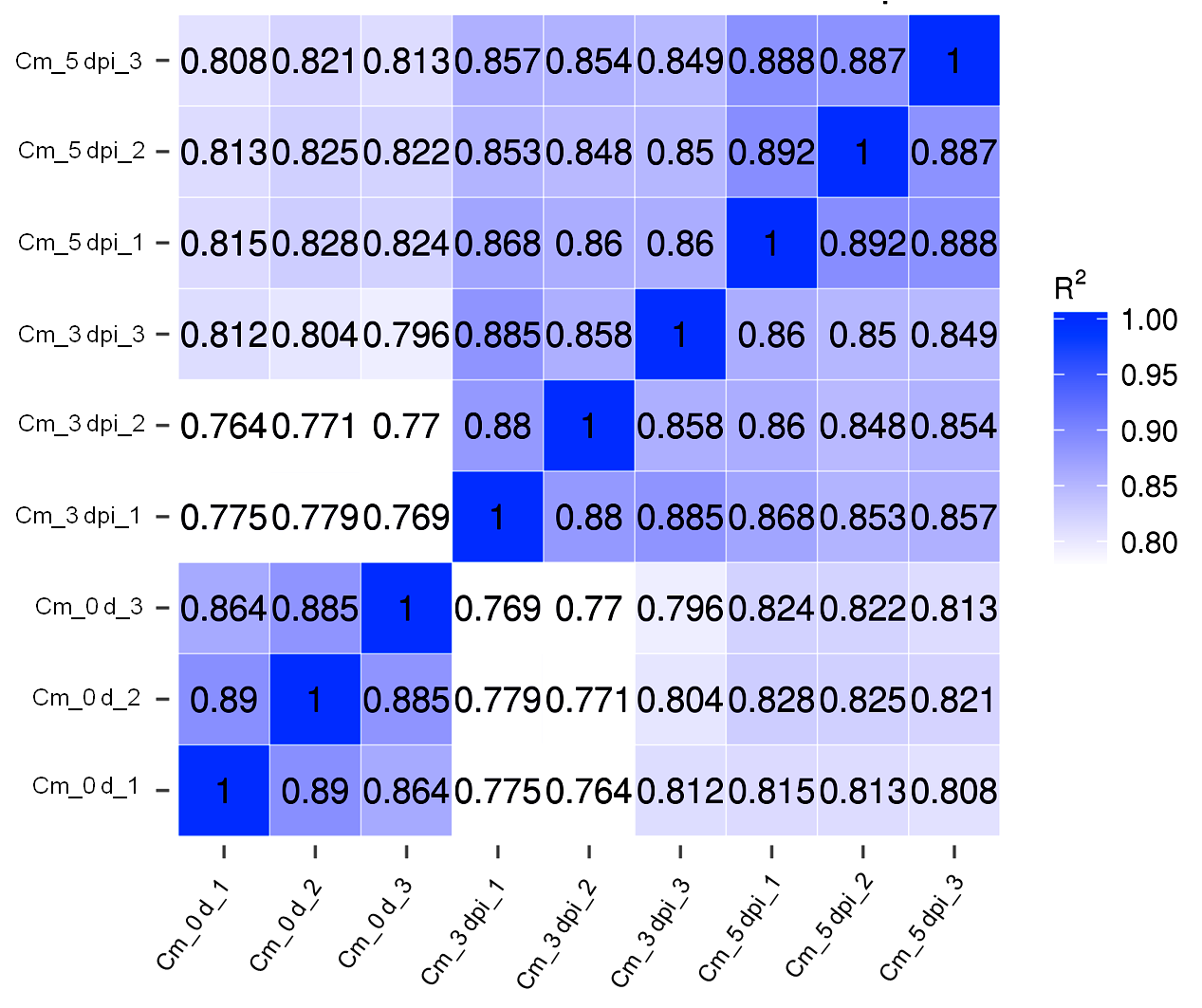


**Supplementary Fig. S2** Pearson Correlation between samples. R^2^ represents determination coefficient, and the darker the colour, the stronger the correlation. R-Studio v8.8.171971 was used to calculate Pearson Correlation between samples. 0.8 ≤ R^2^ ≤ 1.0 represents the highest correlation; 0.6 ≤ R^2^ < 0.8 represents higher correlation; 0.4 ≤ R^2^ < 0.6 represents middle correlation; 0.2 ≤ R^2^ < 0.4 represents lower correlation; 0 ≤ R^2^ < 0.2 represents the lowest correlation or no correlation.


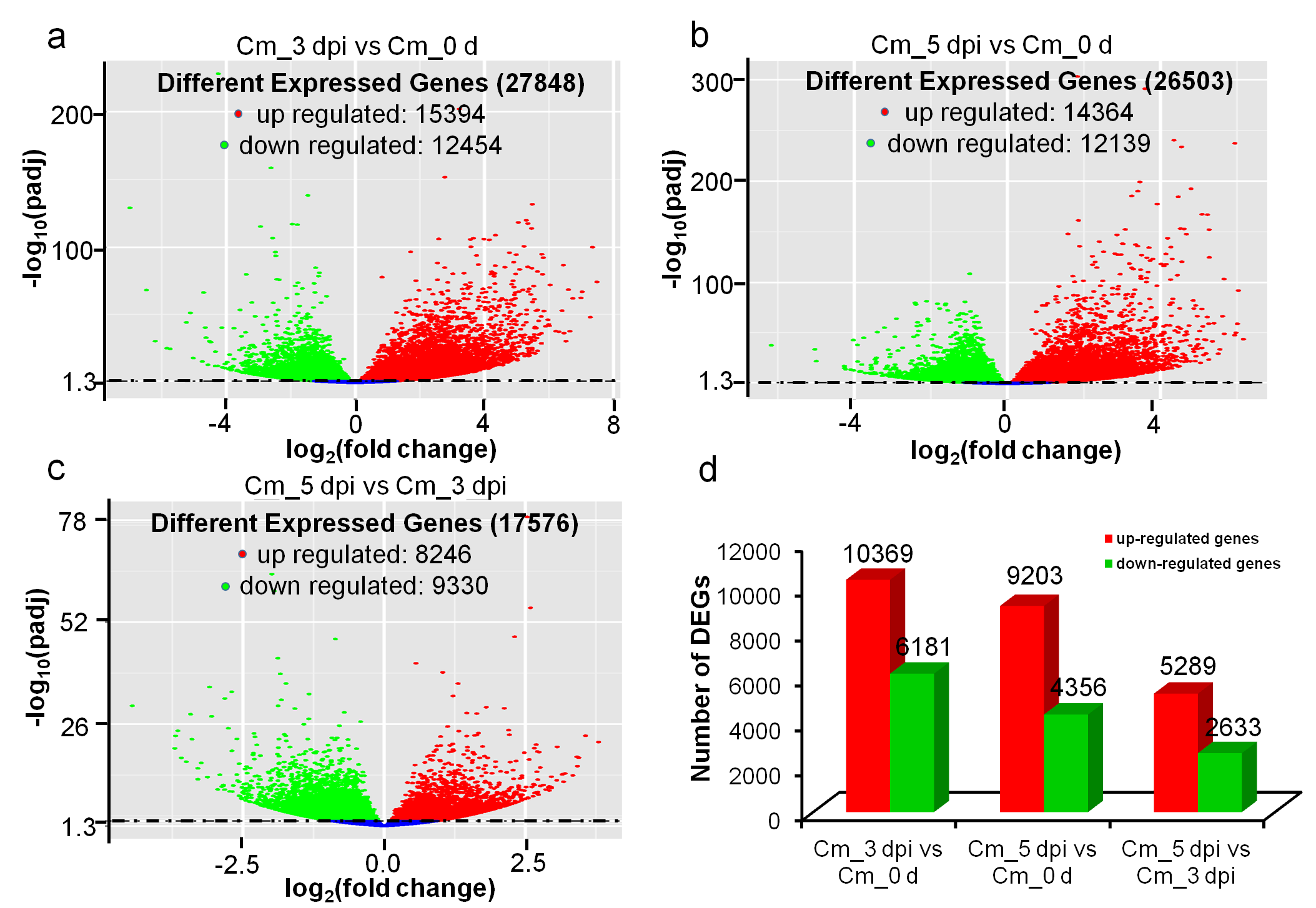


**Supplementary Fig. S3** Different expressed genes (DEGs) in Cm_3 dpi vs Cm_0 d, Cm_5 dpi vs Cm_0 d and Cm_5 dpi vs Cm_3 dpi. a, b and c. The volcano plot analysis of DEGs between Cm_3 dpi vs Cm_0 d, Cm_5 dpi vs Cm_0 d and Cm_5 dpi vs Cm_3 dpi; d. The histogram analysis of DEGs between Cm_3 dpi vs Cm_0 d, Cm_5 dpi vs Cm_0 d and Cm_5 dpi vs Cm_3 dpi.


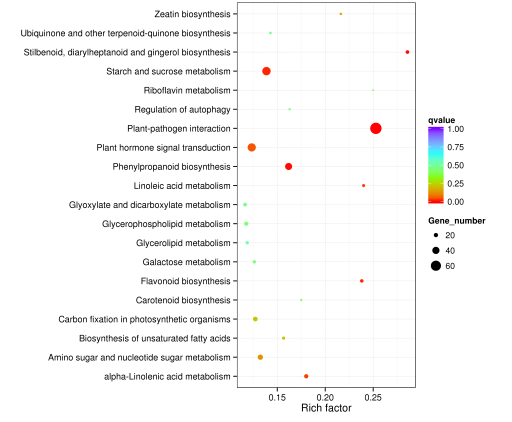


**Supplementary Fig. S4** KEGG pathway enrichment analysis of continuous up-regulated DEGs. The enrichment factor indicates the ratio of DEGs enriched in this pathway to the total number of annotated unigenes. The size and color of each point represents the number of genes enriched in a particular pathway and the *q*-values, respectively. A larger enrichment factor value and lower *q*-values shows a greater degree of enrichment.


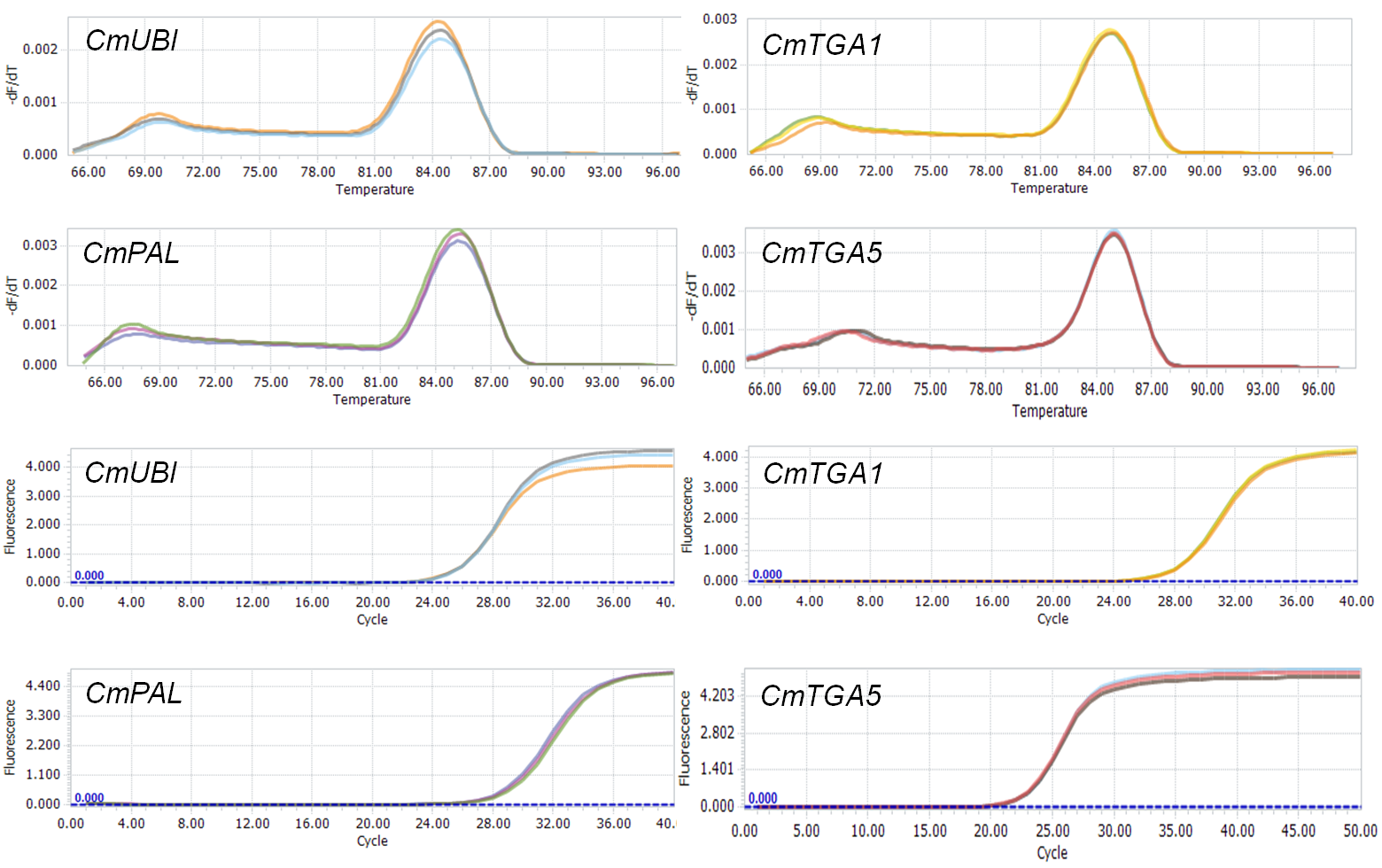


**Supplementary Fig. S5** ‘Melting Peaks’ and ‘Amplification Curves’ of [representive](javascript:;) qPCR amplicons.


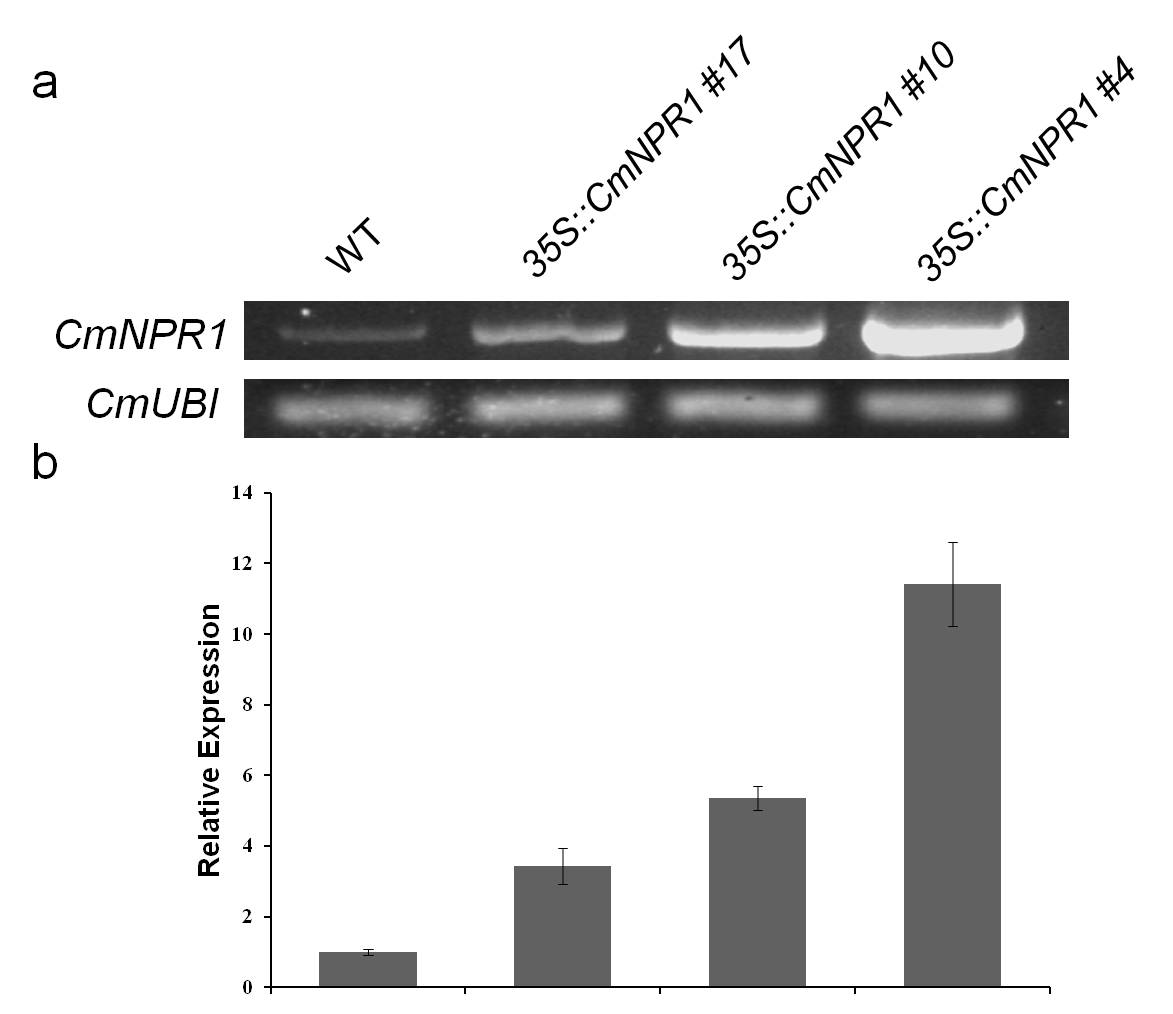


**Supplementary Fig. S6** Relative expression of *CmNPR1* in transformation plants of ‘Huaiju 2^#^’.


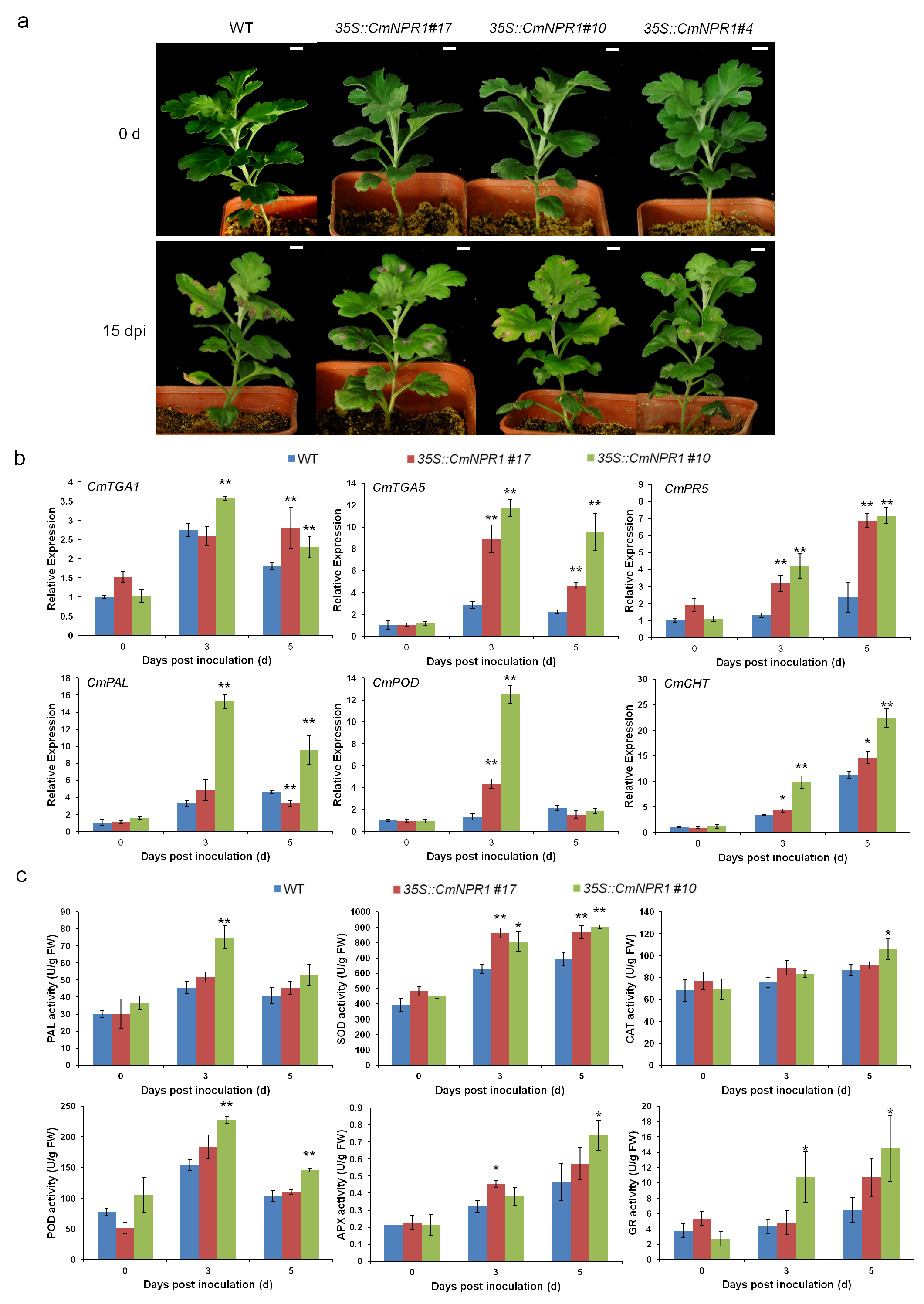


**Supplementary Fig. S7** Overexpression of *CmNPR1* increased ‘Huaiju 2^#^’ resistance to black spot. a. Effects of *Alternaria* sp. infection on phenotype of WT and *CmNPR1* overexpressed plants; b. Effects of *Alternaria* sp. infection on key genes of SA-response pathway and defense gene expression in WT and *CmNPR1* overexpressed plants; c. Effects of *Alternaria* sp. infection on defense enzyme activity in WT and *CmNPR1* overexpressed plants. Data are the means of three repeats. Error bars indicate SEs. Asterisks represent significant differences between WT and *35S::CmNPR1 #17* or *35S::CmNPR1 #10* (Student’s t-test, ***P* < 0.01, **P* < 0.05).


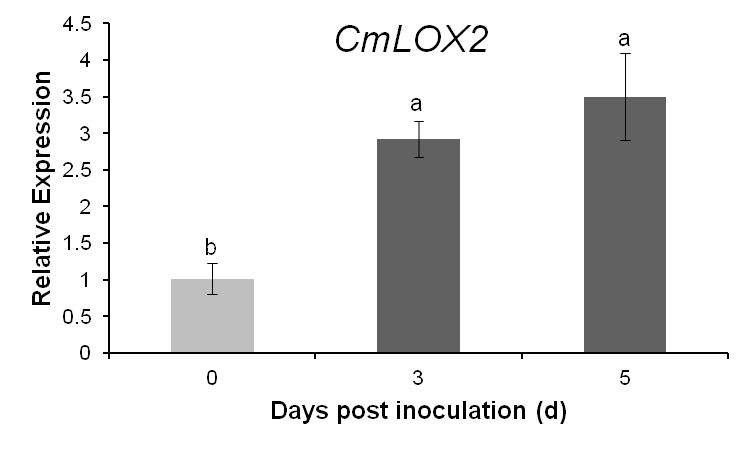


**Supplementary Fig. S8** Expression profile of *CmLOX2* in ‘Huaiju 2^#^’ after inoculation with *Alternaria* sp.

**Supplementary Table S1 Special primers used for qPCR**

| Gene ID | Functional annotation | Primer sequence (forward/reverse 5'to 3') |
| --- | --- | --- |
| c74638_g1 | Peroxidase (*POD*) | F:TTTGTTCTCAGAATAAACCAACTG |
|  |  | R:CCATATCGCTGAACGAAAGAAT |
| c71487_g2 | Phenylalanine ammonia-lyase (*PAL*) | F:CGATTTGTAAGGGAAGAGCT |
|  |  | R:GATCGGAAGAGGAACACCAT |
| c48504_g1 | Glucan endo-1, 3-β-glucosidases (*GLU*) | F:CCTTCCACATGATCCAACAAA |
|  |  | R:AAAGGGCAATGACAGAAACAGA |
| c91107_g1 | Chitinase (*CHT*) | F:TTGTTGGCGATTACCTTGC |
|  |  | R:TTTGCCTCTGCTCATCATCTT |
| c81720_g1 | NPR1 gene | F:GTCCTCTTATTCCATCGTGCC |
|  |  | R:CAAGTCAGTTTCAATCAAAAGGTT |
| c66306_g1 | Pathogenesis-related protein 1 (*PR-1*) | F:CCTTAAACCCAATCTCACAATG |
|  |  | R:TGAGTGCGTTGGTGGAGTT |
| c69081_g1 | Transcription factor MYC2 | F:TAACAAGAAGAACCACCCTGC |
|  |  | R:TGAGAGTACAATCGACTACCCATC |
| c93630_g2 | Disease resistance protein Cf-9 | F:AGTTTGAGGATTCGGGTGTAA |
|  |  | R:ACCGCATTTGGTTGTTGTAC |
| c63892_g1 | Cyclic nucleotide gated channel (*CNGF*) | F:GGCAGCGTTGTTGGAGGAG |
|  |  | R:GGCAAACCTAGAAGCATAGATGG |
| c93335_g1 | Respiratory burst oxidase (*Rboh*) | F:GGTGATGATTCAGTGGCAGTA |
|  |  | R:CCTCCGAAACTTGCCTAAT |
| c93060_g1 | Calcium-dependent protein kinase (*CPK*) | F:TAGTAAAGACCCGACAGAGGA |
|  |  | R:TGCTTCATTTCCTGCTCAA |
| c83997_g1 | Jasmonate ZIM domain-containing protein (*JAZ*) | F:TTTTACAGTGAATTCGCCACCA |
|  |  | R:AGCAGAAGTAGCCTCGTTTGTT |
| *CmUBI* | Reference genes | F:AGCTGAGCAGACTCCCGATG |
|  |  | R:AGGCGAATCATCAGTACCAAGT |

**Supplementary Table S2 Output quality in data**

| Sample | Raw Reads | Clean reads (Percentage/%) | Clean bases | Error (%) | Q20 (%) | Q30 (%) | GC (%) |
| --- | --- | --- | --- | --- | --- | --- | --- |
| Cm_0 d_1 | 58354336 | 56583406 (96.97) | 7.07G | 0.03 | 94.99 | 90.26 | 42.90 |
| Cm_0 d_2 | 65370170 | 62933114 (96.27) | 7.87G | 0.03 | 95.09 | 90.46 | 42.78 |
| Cm_0 d_3 | 54945680 | 53182862 (96.79) | 6.65G | 0.03 | 95.40 | 91.00 | 42.72 |
| Cm_3 dpi_1 | 64285332 | 62100372 (96.60) | 7.76G | 0.03 | 94.81 | 89.92 | 42.57 |
| Cm_3 dpi_2 | 48918962 | 47401094 (96.90) | 5.93G | 0.03 | 95.14 | 90.50 | 42.30 |
| Cm_3 dpi_3 | 65024734 | 62287656 (95.79) | 7.79G | 0.03 | 94.23 | 88.94 | 42.95 |
| Cm_5 dpi_1 | 67989816 | 66060304 (97.16) | 8.26G | 0.03 | 95.03 | 90.29 | 42.46 |
| Cm_5 dpi_2 | 60174876 | 58149022 (96.63) | 7.27G | 0.03 | 94.83 | 89.98 | 42.26 |
| Cm_5 dpi_3 | 59347830 | 57499778 (96.89) | 7.19G | 0.03 | 94.84 | 89.96 | 42.19 |

**Supplementary Table S3 Statistics and analysis of unigenes expression level**

| Sample name | Total mapped  (Percentage/%) | FPKM Interval | | | | | |
| --- | --- | --- | --- | --- | --- | --- | --- |
|  |  | 0-0.1  (Percentage/%) | 0.1-0.3  (Percentage/%) | 0.3-3.57  (Percentage/%) | 3.57-15  (Percentage/%) | 15-60  (Percentage/%) | >60  (Percentage  /%) |
| Cm_0 d_1 | 40274864  (71.18) | 71749  (44.22) | 12369  (7.62) | 44932  (27.69) | 19802  (12.20) | 10302  (6.35) | 3098  (1.91) |
| Cm_0 d_2 | 44193966  (70.22) | 66065  (40.72) | 13801  (8.51) | 49211  (30.33) | 19709  (12.15) | 10397  (6.41) | 3069  (1.89) |
| Cm_0 d_3 | 37554046  (70.61) | 69203  (42.65) | 11773  (7.26) | 47284  (29.14) | 20180  (12.44) | 10719  (6.61) | 3093  (1.91) |
| Cm_3 dpi_1 | 43446446  (69.96) | 71799  (44.25) | 13761  (8.48) | 45794  (28.22) | 18273  (11.26) | 9263  (5.71) | 3362  (2.07) |
| Cm_3 dpi_2 | 32682712  (68.95) | 70572  (43.50) | 10526  (6.49) | 47311  (29.16) | 20067  (12.37) | 10323  (6.36) | 3453  (2.13) |
| Cm_3 dpi_3 | 43901810  (70.48) | 69640  (42.92) | 14077  (8.68) | 47033  (28.99) | 18973  (11.69) | 9515  (5.86) | 3014  (1.86) |
| Cm_5 dpi_1 | 45693724  (69.17) | 61702  (38.03) | 13923  (8.58) | 51477  (31.73) | 20395  (12.57) | 11279  (6.95) | 3476  (2.14) |
| Cm_5 dpi_2 | 39966538  (68.73) | 64220  (39.58) | 12279  (7.57) | 49980  (30.80) | 20752  (12.79) | 11513  (7.10) | 3508  (2.16) |
| Cm_5 dpi_3 | 39080764  (67.97) | 62367  (38.44) | 11835  (7.29) | 51521  (31.75) | 21238  (13.09) | 11700  (7.21) | 3591  (2.21) |

**Supplementary Table S4 Disease resistance related differentially expressed genes**

| **Gene ID** | **Annotion** | **Cm_3 dpi vs Cm_0 d** | | **Cm_5 dpi vs Cm_0 d** | |
| --- | --- | --- | --- | --- | --- |
|  |  | **log2FC** | **Padj** | **log2FC** | **Padj** |
| c92981_g1 | BRI1-associated kinase 1, BAK1 | 1.5 | 1.02E-07 |  |  |
| c89846_g2 | BRI1-associated kinase 1, BAK1 | 3.4 | 2.08E-16 | 3.4 | 1.49E-36 |
| c94216_g1 | BRI1-associated kinase 1, BAK1 | 1.7 | 6.87E-19 |  |  |
| c90652_g1 | BRI1-associated kinase 1, BAK1 | 1.6 | 2.30E-25 |  |  |
| c90189_g6 | BRI1-associated kinase 1, BAK1 | 1.7 | 0.0084696 |  |  |
| c80409_g1 | BRI1-associated kinase 1, BAK1 | 1.7 | 2.88E-17 |  |  |
| c79359_g1 | BRI1-associated kinase 1, BAK1 | 1.6 | 1.01E-21 |  |  |
| c74149_g1 | BRI1-associated kinase 1, BAK1 | 2.8 | 1.85E-19 | 2.9 | 6.27E-44 |
| c93605_g1 | Chitin elicitor receptor kinase 1, CERK1 | 1.7 | 1.45E-25 |  |  |
| c81335_g1 | Chitin elicitor receptor kinase 1, CERK1 | 1.6 | 5.07E-15 |  |  |
| c62667_g1 | Chitin elicitor receptor kinase 1, CERK1 | 1.9 | 2.32E-20 |  |  |
| c46925_g2 | Chitin elicitor receptor kinase 1, CERK1 | 3.3 | 1.09E-07 | 1.9 | 0.0012137 |
| c93335_g2 | Respiratory burst oxidase D, *RbohD* | 4.2 | 5.32E-81 | 3.0 | 4.94E-67 |
| c93335_g1 | Respiratory burst oxidase C, *RbohC* | 4.3 | 2.59E-79 | 3.2 | 4.82E-78 |
| c89350_g1 | Pathogen-inducible α-dioxygenases | 1.3 | 0.00075449 |  |  |
| c91107_g1 | Chitinase, CHT | 2.2 | 1.34E-09 | 2.4 | 8.87E-30 |
| c88590_g1 | Chitinase, CHT | 2.4 | 1.16E-30 | 1.9 | 8.70E-27 |
| c87690_g2 | Chitinase, CHT | 4.2 | 5.84E-49 | 3.3 | 9.89E-45 |
| c87690_g1 | Chitinase, CHT | 3.8 | 7.04E-33 | 3.1 | 5.40E-89 |
| c82424_g3 | Chitinase, CHT | 2.1 | 5.76E-06 | 2.6 | 1.07E-23 |
| c71470_g1 | Chitinase, CHT | 2.6 | 1.35E-08 | 2.9 | 7.65E-25 |
| c67031_g1 | Chitinase, CHT | 1.9 | 2.97E-06 |  |  |
| c57330_g1 | Chitinase, CHT |  |  | 3.8 | 3.17E-12 |
| c88875_g1 | Glucan endo-1, 3-β-glucosidases, GLU | 2.8 | 2.75E-21 | 1.9 | 1.37E-33 |
| c86420_g3 | Glucan endo-1, 3-β-glucosidases, GLU | 1.6 | 2.88E-14 |  |  |
| c84861_g1 | Glucan endo-1, 3-β-glucosidases, GLU | 7.0 | 2.37E-63 | 5.2 | 9.39E-31 |
| c75920_g1 | Glucan endo-1, 3-β-glucosidases, GLU | 1.8 | 0.0024171 |  |  |
| c72540_g1 | Glucan endo-1, 3-β-glucosidases, GLU | 2.3 | 1.12E-06 | 2.9 | 3.96E-14 |
| c69780_g2 | Glucan endo-1, 3-β-glucosidases, GLU | 2.6 | 2.67E-17 | 1.8 | 7.67E-25 |
| c69244_g1 | Glucan endo-1, 3-β-glucosidases, GLU | 1.6 | 0.011589 | 3.9 | 2.90E-22 |
| c67862_g1 | Glucan endo-1, 3-β-glucosidases, GLU | 2.0 | 0.0022044 | 1.7 | 0.002604 |
| c48504_g1 | Glucan endo-1, 3-β-glucosidases, GLU | 2.0 | 0.0001276 | 1.9 | 0.0001288 |
| c35279_g1 | Glucan endo-1, 3-β-glucosidases, GLU |  |  | 1.5 | 0.010352 |
| c91099_g1 | Peroxidase, POD | 4.1 | 1.16E-23 | 2.1 | 9.28E-07 |
| c89797_g2 | Peroxidase, POD |  |  | 1.8 | 2.41E-05 |
| c89432_g5 | Peroxidase, POD | 5.1 | 1.10E-30 | 4.3 | 9.49E-27 |
| c89242_g2 | Peroxidase, POD | 3.4 | 5.22E-08 | 1.8 | 0.0022888 |
| c86692_g1 | Peroxidase, POD | 4.5 | 3.31E-14 | 3.5 | 6.81E-12 |
| c83414_g4 | Peroxidase, POD | 1.5 | 0.0093678 | 1.3 | 0.0011589 |
| c79194_g7 | Peroxidase, POD | 4.3 | 3.66E-17 | 3.9 | 2.49E-20 |
| c74638_g1 | Peroxidase, POD | 4.7 | 1.36E-32 | 3.1 | 2.91E-32 |
| c74311_g2 | Peroxidase, POD | 1.9 | 0.0030086 | 3.0 | 5.85E-10 |
| c69523_g2 | Peroxidase, POD | 4.5 | 2.29E-21 | 2.9 | 2.09E-17 |
| c69523_g1 | Peroxidase, POD | 4.6 | 6.10E-14 | 3.7 | 7.84E-12 |
| c65618_g1 | Peroxidase, POD | 5.1 | 1.60E-27 | 4.3 | 8.82E-29 |
| c90494_g1 | Peroxidase, POD |  |  | 2.1 | 8.03E-39 |
| c89797_g1 | Peroxidase, POD |  |  | 1.7 | 0.00020939 |
| c77607_g1 | Peroxidase, POD |  |  | 1.7 | 0.00093926 |
| c84289_g1 | Polyphenol oxidase, PPO | 2.9 | 5.79E-11 | 1.7 | 9.87E-05 |
| c56866_g1 | Polyphenol oxidase, PPO | 2.1 | 5.15E-07 | 2.0 | 1.22E-09 |
| c59894_g1 | Polyphenol oxidase, PPO |  |  | 1.6 | 0.0016933 |
| c51052_g1 | Polyphenol oxidase, PPO |  |  | 1.9 | 0.00071439 |
| c90823_g2 | Phenylalanine ammonia-lyase, PAL | 4.3 | 2.50E-42 | 2.6 | 2.24E-18 |
| c90823_g1 | Phenylalanine ammonia-lyase, PAL | 2.5 | 1.03E-24 |  |  |
| c84765_g3 | Phenylalanine ammonia-lyase, PAL | 1.8 | 2.94E-06 |  |  |
| c84765_g2 | Phenylalanine ammonia-lyase, PAL | 2.0 | 1.08E-08 |  |  |
| c84765_g1 | Phenylalanine ammonia-lyase, PAL | 2.0 | 2.78E-09 |  |  |
| c67434_g1 | Phenylalanine ammonia-lyase, PAL | 1.8 | 1.25E-06 |  |  |
| c93060_g1 | Calcium-dependent protein kinase, CPK | 4.2 | 8.83E-79 | 4.8 | 1.42E-52 |
| c92715_g1 | Calcium-dependent protein kinase, CPK | 3.4 | 1.41E-46 | 3.7 | 9.71E-25 |
| c89916_g1 | Calcium-dependent protein kinase, CPK | 1.9 | 2.55E-72 | 2.1 | 5.75E-14 |
| c89595_g2 | Calcium-dependent protein kinase, CPK |  |  | 1.8 | 7.18E-16 |
| c87235_g1 | Calcium-dependent protein kinase, CPK | 1.7 | 5.69E-24 | 2.2 | 1.07E-25 |
| c86224_g3 | Calcium-dependent protein kinase, CPK | 1.7 | 3.99E-34 | 2.1 | 6.41E-30 |
| c86224_g1 | Calcium-dependent protein kinase, CPK | 1.5 | 8.82E-06 | 2.4 | 9.89E-13 |
| c82769_g1 | Calcium-dependent protein kinase, CPK | 3.5 | 1.02E-127 | 4.2 | 2.95E-106 |
| c78350_g1 | Calcium-dependent protein kinase, CPK |  |  | 1.6 | 3.47E-07 |
| c77914_g1 | Calcium-dependent protein kinase, CPK | 1.9 | 7.65E-24 | 2.8 | 3.69E-28 |
| c74478_g1 | Calcium-dependent protein kinase, CPK | 2.5 | 5.08E-62 | 2.7 | 3.84E-15 |
| c65441_g3 | Calcium-dependent protein kinase, CPK | 1.7 | 0.00039453 | 3.2 | 1.27E-11 |
| c63138_g1 | Calcium-dependent protein kinase, CPK | 1.5 | 2.23E-18 | 2.4 | 1.75E-30 |
| c52966_g1 | Calcium-dependent protein kinase, CPK |  |  | 2.2 | 0.00023533 |
| c93060_g1 | Calcium-dependent protein kinase, CPK | 4.2 | 8.83E-79 |  |  |
| c73057_g2 | Calcium-dependent protein kinase, CPK | 2.3 | 3.33E-06 | 3.6 | 6.87E-12 |
| c88103_g2 | Calcium-dependent protein kinase, CPK |  |  | 2.2 | 2.31E-42 |
| c82588_g2 | Calcium-dependent protein kinase, CPK |  |  | 1.6 | 0.00083564 |
| c77914_g2 | Calcium-dependent protein kinase, CPK | 3.3 | 1.96E-74 | 4.0 | 8.96E-68 |
| c86224_g2 | Calcium-dependent protein kinase, CPK | 1.8 | 1.21E-07 | 2.8 | 1.51E-21 |
| c89595_g4 | Calcium-dependent protein kinase, CPK |  |  | 2.2 | 1.07E-25 |
| c65441_g2 | Calcium-dependent protein kinase, CPK |  |  | 1.5 | 1.20E-14 |
| c89340_g2 | Calmodulin, CALM | 2.0 | 1.61E-07 | 2.1 | 6.76E-10 |
| c88601_g2 | Calmodulin, CALM | 2.5 | 4.62E-05 |  |  |
| c76325_g3 | Calmodulin, CALM | 2.6 | 3.44E-20 | 2.2 | 1.31E-17 |
| c72242_g1 | Calmodulin, CALM | 2.0 | 2.54E-08 | 1.6 | 6.29E-20 |
| c71168_g1 | Calmodulin, CALM | 2.9 | 5.63E-12 |  |  |
| c65461_g1 | Calmodulin, CALM | 2.9 | 2.96E-11 | 2.7 | 1.10E-13 |
| c59940_g1 | Calmodulin, CALM |  |  | 4.4 | 2.26E-33 |
| c66508_g1 | Calmodulin, CALM |  |  | 1.9 | 1.07E-09 |
| c89588_g2 | Calcium-binding protein CML | 2.3 | 4.78E-12 |  |  |
| c86045_g2 | Calcium-binding protein CML | 3.0 | 1.75E-10 | 1.6 | 0.00047964 |
| c85287_g3 | Calcium-binding protein CML | 2.3 | 3.35E-19 | 1.9 | 1.98E-49 |
| c82008_g5 | Calcium-binding protein CML | 2.1 | 5.22E-05 |  |  |
| c79549_g3 | Calcium-binding protein CML | 2.8 | 4.46E-08 |  |  |
| c79549_g1 | Calcium-binding protein CML | 3.9 | 2.18E-14 | 2.3 | 1.07E-06 |
| c78429_g2 | Calcium-binding protein CML | 1.8 | 1.52E-05 |  |  |
| c77903_g1 | Calcium-binding protein CML | 2.5 | 4.93E-18 | 2.2 | 8.80E-24 |
| c75155_g1 | Calcium-binding protein CML | 3.4 | 1.76E-12 | 2.9 | 1.47E-15 |
| c71511_g1 | Calcium-binding protein CML | 2.1 | 1.13E-06 | 2.3 | 4.27E-12 |
| c68541_g2 | Calcium-binding protein CML | 2.8 | 1.51E-12 | 1.8 | 2.57E-06 |
| c66508_g1 | Calcium-binding protein CML |  |  | 1.9 | 1.07E-09 |
| c63174_g1 | Calcium-binding protein CML | 4.0 | 1.07E-14 | 2.1 | 5.45E-05 |
| c89340_g2 | Calcium-binding protein CML | 2.0 | 1.61E-07 | 2.1 | 6.76E-10 |
| c79549_g2 | Calcium-binding protein CML | 1.9 | 0.0021011 | 1.7 | 0.00064828 |
| c76325_g3 | Calcium-binding protein CML | 2.6 | 3.44E-20 | 2.2 | 1.31E-17 |
| c65461_g1 | Calcium-binding protein CML | 2.9 | 2.96E-11 | 2.7 | 1.10E-13 |
| c92620_g1 | Cyclic nucleotide gated channel, CNGF | 1.7 | 5.29E-12 | 2.1 | 1.11E-20 |
| c81362_g2 | Cyclic nucleotide gated channel, CNGF | 2.6 | 1.83E-22 | 2.0 | 7.60E-26 |
| c78282_g1 | Cyclic nucleotide gated channel, CNGF |  |  | 1.5 | 7.34E-16 |
| c70986_g1 | Cyclic nucleotide gated channel, CNGF | 2.1 | 2.05E-19 | 2.3 | 7.99E-15 |
| c64658_g2 | Cyclic nucleotide gated channel, CNGF | 3.6 | 3.21E-12 |  |  |
| c64658_g1 | Cyclic nucleotide gated channel, CNGF | 3.8 | 2.82E-14 | 1.9 | 0.00010542 |
| c63892_g1 | Cyclic nucleotide gated channel, CNGF | 3.8 | 6.20E-17 | 1.4 | 0.0018571 |
| c61927_g1 | Cyclic nucleotide gated channel, CNGF | 1.8 | 2.12E-13 | 2.2 | 3.46E-14 |
| c81362_g1 | Cyclic nucleotide gated channel, CNGF | 2.7 | 3.34E-26 | 2.0 | 8.45E-48 |
| c70986_g1 | Cyclic nucleotide gated channel, CNGF | 2.1 | 2.05E-19 |  |  |
| c90637_g1 | NPR1 gene | 1.7 | 2.39E-26 |  |  |
| c70940_g1 | Pathogenesis-related protein, PR | 1.8 | 3.06E-05 | 2.2 | 1.77E-09 |
| c80721_g1 | Pathogenesis-related protein, PR | 2.2 | 3.10E-15 |  |  |
| c70867_g1 | Pathogenesis-related protein, PR | 7.5 | 3.02E-75 | 6.0 | 8.41E-93 |
| c63071_g1 | Pathogenesis-related protein, PR | 1.8 | 1.35E-06 |  |  |
| c66306_g1 | Pathogenesis-related protein, PR |  |  | 1.6 | 3.68E-05 |
| c75701_g1 | Transcription factor TGA | 1.7 | 0.0060164 |  |  |
| c93811_g1 | Lipoxygenase, LOX | 2.9 | 6.94E-46 | 1.9 | 3.45E-42 |
| c86279_g1 | Lipoxygenase, LOX | 2.0 | 2.35E-24 | 1.5 | 9.05E-25 |
| c84020_g3 | Lipoxygenase, LOX | 2.4 | 7.02E-05 |  |  |
| c84020_g2 | Lipoxygenase, LOX | 4.2 | 4.88E-57 | 2.8 | 1.36E-55 |
| c81040_g3 | Lipoxygenase, LOX | 2.1 | 8.64E-42 | 1.7 | 3.90E-36 |
| c91403_g1 | Allene oxide synthase, AOS | 1.7 | 3.60E-15 |  |  |
| c93397_g1 | 12-oxophytodienoic acid reductase, OPR | 2.1 | 3.44E-37 |  |  |
| c92242_g4 | 12-oxophytodienoic acid reductase, OPR | 2.3 | 6.75E-09 |  |  |
| c92242_g3 | 12-oxophytodienoic acid reductase, OPR | 2.2 | 0.00032961 |  |  |
| c65958_g1 | 12-oxophytodienoic acid reductase, OPR | 2.2 | 4.72E-27 | 1.5 | 5.97E-31 |
| c63778_g1 | 12-oxophytodienoic acid reductase, OPR | 2.2 | 3.97E-07 | 1.7 | 0.00038905 |
| c57510_g1 | 12-oxophytodienoic acid reductase, OPR | 2.1 | 5.34E-06 |  |  |
| c27114_g1 | Jasmonic acid-amino synthetase, JAR1 | 3.3 | 6.00E-08 | 2.8 | 2.82E-07 |
| c20889_g1 | Jasmonic acid-amino synthetase, JAR1 | 2.3 | 0.00039876 |  |  |
| c83997_g1 | JAZ gene | 4.2 | 6.91E-14 | 3.2 | 1.05E-47 |
| c83926_g1 | JAZ gene | 2.4 | 2.26E-08 | 1.6 | 5.82E-09 |
| c68101_g1 | JAZ gene | 1.5 | 0.018297 |  |  |
| c88402_g1 | Transcription factor MYC2 | 2.4 | 3.28E-13 |  |  |
| c69081_g1 | Transcription factor MYC2 | 2.3 | 2.67E-12 |  |  |
| c63085_g1 | Transcription factor MYC2 | 2.3 | 4.18E-12 |  |  |
| c86524_g2 | Transcription factor MYC2 | 1.8 | 8.18E-09 |  |  |
| c90909_g3 | Mitogen-activated protein kinase 6, MPK6 | 5.2 | 2.54E-43 | 4.8 | 3.70E-193 |
| c92329_g1 | Mitogen-activated protein kinase 3, MPK3 | 5.0 | 2.82E-46 | 4.5 | 1.90E-234 |
| c80122_g1 | WRKY transcription factor 33 | 7.3 | 3.44E-49 | 5.6 | 8.84E-35 |
| c88477_g4 | WRKY transcription factor 33 | 6.6 | 7.97E-39 | 5.1 | 2.44E-29 |
| c85674_g1 | WRKY transcription factor 33 | 5.8 | 4.32E-93 | 5.1 | 5.33E-168 |
